# Folate and global health: part 5. syntheses on neuropsychiatric disorders

**DOI:** 10.7189/jogh.16.04286

**Published:** 2026-08-17

**Authors:** Samantha Yoo, Azita Montazeri, Derrick Bennett, Yacong Bo, Peizhan Chen, Susan Duthie, Natalie Jensen, Atipatsa Kaminga, Jun-Shi Lai, Xue Li, Amanda J MacFarlane, Homero Martinez, Helene McNulty, Franco Momoli, Peter Mossey, Ron Munger, Rajendra Prasad Parajuli, Monique Potvin Kent, Michele Rubini, Marjanne Senekal, Lindsey Sikora, Alain Stintzi, Evropi Theodoratou, Hui Wang, Ann Yaktine, Julian Little

**Affiliations:** 1 School of Epidemiology and Public Health, Faculty of Medicine, University of Ottawa, Canada; 2 Nuffield Department of Population Health, University of Oxford, UK; 3 School of Public Health, Xinxiang Medical University, China; 4 Clinical Research Centre, Ruijin Hospital, Shanghai Jiao Tong University School of Medicine, Shanghai, China; 5 School of Pharmacy and Life Sciences, Robert Gordon University, Scotland, UK; 6 Department of Mathematics and Statistics, Faculty of Science, Technology and Innovation, Mzuzu University, Malawi; 7 Singapore Institute for Clinical Sciences, Agency for Science, Technology and Research, Singapore; 8 Centre for Population Health Sciences, University of Edinburgh, UK; 9 Nutrition Research Division, Health Canada, Canada; 10 Research and Development Unit, Nutrition International, Canada; 11 Nutrition Innovation Centre for Food and Health, School of Biomedical Sciences, Ulster University, Coleraine, Northern Ireland, UK; 12 School of Dentistry, University of Dundee, Scotland, UK; 13 Department of Nutrition, Dietetics, and Food Sciences, College of Agriculture and Applied Sciences, Utah State University, USA; 14 Herbert Wertheim School of Public Health and Human Longevity Science, University of California San Diego, USA; 15 Department of Neuroscience and Rehabilitation, University of Ferrara, Italy; 16 Department of Human Biology, Faculty of Health Sciences, University of Cape Town, South Africa; 17 Health Sciences Library, University of Ottawa, Canada; 18 School of Pharmacy, Faculty of Medicine, University of Ottawa, Canada; 19 Centre for Global Health, Usher Institute, College of Medicine and Veterinary Medicine, University of Edinburgh, UK; 20 School of Public Health, Faculty of Medicine, Shanghai Jiao Tong University, China; 21 Food and Nutrition Board, Health and Medicine Division, National Academies of Sciences, Engineering, and Medicine, USA

**Keywords:** folate, folic acid, neuropsychiatric, umbrella review, evidence synthesis

## Abstract

**Background:**

The global burden of neuropsychiatric disorders has been increasing rapidly. Folate has been studied in the context of these disorders due to its role in cellular methylation capacity, which is crucial for normal neuronal gene expression and neurotransmitter syntheses. Epidemiological evidence linking folate and neuropsychiatric disorders is growing, yet inconclusive.

**Methods:**

We searched MEDLINE, Embase, CINAHL, the Cochrane Library, and the Database of Abstracts of Reviews of Effects from inception to February 2024 for systematic reviews and meta-analyses investigating the associations between folate exposure and any neuropsychiatric disorders. Two independent reviewers screened the syntheses in two stages and extracted relevant data. Evidence was categorised into unique associations (unique exposure measure – unique outcome – unique setting). We identified evidence for each category of unique associations. We evaluated the risk of bias of all included syntheses using ROBIS and assessed the credibility of evidence using predefined criteria.

**Results:**

From a total of 76 syntheses, we identified 44 unique associations from meta-analyses and 18 unique associations from qualitative syntheses across 14 outcome categories. Most of the reviews consisted of case-control studies or cross-sectional studies, and most were at high risk of bias. We identified two associations at a suggestive level of credibility: folic acid supplementation during or before pregnancy was associated with a reduced risk of perinatal depression, while prenatal maternal folic acid use was associated with a reduced risk of autism spectrum disorder in offspring. Seven associations were downgraded to weak due to unavailable data. Other associations were at a weak level of credibility due to insufficient statistical power.

**Conclusions:**

The available evidence on the relationship between folate status and neuropsychiatric disorders primarily comprised data from observational studies, limiting causal inference on folate exposure and the risk of onset or progression of neuropsychiatric disorders. Prospective studies and sex-stratified analyses could add to the evolving knowledge on this topic.

**Registration:**

PROSPERO: CRD42021265041

Neurological disorders have one of the highest morbidity rates globally and are leading cause of disability-adjusted life-years (DALYs), with Alzheimer’s disease (AD), other types of dementia, Parkinson’s disease (PD), and epilepsy each accounting for over 10 million DALYs in 2019 [1]. This burden continues to increase in many countries, in part due to population growth, ageing, and lifestyle risk factors [2,3], as well as rapid urbanisation without planned infrastructure, particularly in less developed countries [4–6]. The global prevalence of mental disorders has also been increasing steadily since the 1990s, resulting in them ranking as the seventh leading cause of DALYs in 2019 [7]. The DALYs from neurological and mental disorders vary substantially by country [7] and sex [2,8], although differential diagnosis rates and life-expectancy may contribute to these variations.

Studies have identified functional overlaps between neurological and psychiatric disorders [9]. For example, mild cognitive impairment, AD, and major depressive disorder share similarities in global brain functional interactions, while epilepsy, attention deficit hyperactivity disorder, and schizophrenia bear similarities in their functional connectivity in the brain [9]. Furthermore, psychiatric symptoms such as hallucinations, delusion, anxiety, and depressive state are commonly experienced by individuals with neurological disorders, such as PD or epilepsy [10–12]. A recent report on the genetic overlaps between these two groups of disorders – that is, shared genomic components underlying both neurological and psychiatric disorders – also supports an integrated approach to these outcomes [13]. Of note are potentially bidirectional links between neuropsychiatric disorders and other chronic conditions, such as cardiovascular diseases, cancer, and respiratory conditions, whereby individuals with neuropsychiatric conditions often suffer physical comorbidities and those with chronic physical conditions suffer from mental, behavioural, or neurological conditions [14–18].

The relationship between folate and neuropsychiatric disorders has been studied based on the former’s role in cellular methylation metabolism, and in turn, in the development of the central nervous system [19] and the synthesis of neurotransmitters [20–22]. Individuals with depression [23,24] and schizophrenia [25,26] have been reported to have lower plasma folate concentrations than healthy controls. While meta-analyses of randomised controlled trials (RCTs) showed that adjunctive folic acid (FA) or methylfolate treatment may be effective in improvement of symptoms in major depressive disorder [27–30], the results for schizophrenia remain inconclusive [27,31].

The evidence on the relationship between folate status and neuropsychiatric disorders is fragmented in terms of the populations studied, folate exposure assessment methods, and the specific neuropsychiatric outcomes considered. For example, there is variation in how folate exposures have been measured and/or outcomes operationalised in specific subgroups. The fact that the studies have been predominantly of cross-sectional or case-control design also make the overall evidence difficult to interpret. Moreover, policy discussions around folate (*e.g.* introduction of mandatory FA fortification) have been focused on a small number of outcomes, *e.g.* balancing the potential benefits of reduction in the prevalence at birth of neural tube defects against the potential harms of masking of vitamin B12 deficiency, and increased risk of colorectal neoplasia. It is important that a broader range of outcomes are considered in policy decision-making, considering the biological potency of FA and the impact on population nutritional exposure. The previous umbrella review on this topic was limited to systematic reviews with meta-analyses in an adult population [32].

In this sense, a comprehensive synthesis of the entirety of the existing evidence – systematic reviews with and without meta-analyses on all outcomes across age groups – with critical appraisal of the quality and credibility of the reported findings could inform researchers and knowledge users. For this purpose, we designed a series of umbrella reviews that use similar methods [33], aiming to provide a balanced understanding of potential benefits and harms associated with folate status in six different categories of health outcomes. Umbrella reviews are a useful approach to identify scientific investigations reported during a specific time period and to assess the volume and quality of the evidence in the form of systematic reviews and meta-analyses [34,35]. They also critically assess the level of confidence in the methodological quality of the evidence to potential interventions at a population level and allow for triangulation of evidence arising from different measurements, populations, and experimental designs.

## METHODS

We conducted an umbrella review of systematic reviews, where we searched MEDLINE and MEDLINE in Process *via* Ovid (1946 to 13 February 2024), Embase Classic + Embase *via* Ovid (1947 to 13 February 2024), CINAHL *via* EBSCOHost (1981 to 13 February 2024), the Cochrane Database of Systematic Reviews (2005 to 28 December 2021), and the Database of Abstracts of Reviews of Effects *via* Ovid (1994 to first quarter of 2016) for systematic reviews, with or without meta-analyses, that investigated associations between folate intake or status (measured as dietary intake, supplementation, or blood concentrations) and any neuropsychiatric outcome. Syntheses examining homocysteine as a marker of folate exposure or those examining multivitamins or multiple nutrients without separate quantification of folate intake were excluded. We did not impose any restrictions on the study population or design of component studies.

Two reviewers (SY, AM, or NJ) independently screened the articles in two stages (title/abstract and full-text) and extracted data using a standardised extraction template comprising six broad sections (Table S2 in the **Online Supplementary Document**): study information (first author, year of publication, year of search, exposure measure, outcome measure, risk of bias assessment); study population (eligibility criteria, countries represented in the review, participants’ age, sex, other sociodemographic features, if any); exposure details (type of exposure measure, method of measurement, time of measurement); outcome details (definition of outcome, measurement tool/scale used), reported qualitative synthesis, and information about components of meta-analysis, if a meta-analysis was reported (number of primary studies, number of total participants, number of cases, reported summary effect, heterogeneity measure, measure of small study effects, methodological quality assessment, dose-response effects, subgroup analyses, if available). All discrepancies were resolved by consensus. Two reviewers (AM, NJ) then independently assessed the included syntheses for methodological quality using the ROBIS tool [36].

We categorised all evidence by type of exposure measure, outcome, and setting (population subgroups or geographical regions) to identify unique associations (unique exposure – unique outcome – unique setting). For each category of unique associations, we examined the evidence for consistencies in direction, magnitude, and statistical significance of the summary effects. If concordant, we identified the evidence with the largest sample size. If discordant, the evidence was selected based on the largest total sample size, the largest number of cases (for binary outcomes), recency of publication, and the highest methodological quality as assessed by ROBIS. Design of the component studies were not part of the identification criteria; however, in cases where the evidence selected for a unique association consisted entirely or predominantly of retrospective studies, we examined evidence in the same category comprising entirely or predominantly prospective investigations (intervention trials, prospective cohort studies, nested case-control studies, or case cohort studies) and compared the findings to see if retrospective design of component studies biased the pooled estimates in any direction.

Lastly, we evaluated the credibility of the selected evidence using predefined criteria (**Table 1**). For unique associations that were assessed to be of a ‘convincing’ level of credibility, we sought, based on the data reported, to re-calculate the summary effects and 95% confidence intervals (CIs); predictive intervals to understand the dispersion of effect sizes [37]; heterogeneity between the studies using *I*^2^ and *P*-value; small study effects using Egger’s test of symmetry [38] with a significance threshold *P* < 0.10; and excess significance [39] with a threshold *P* < 0.10. For all other levels of credibility, we reported the statistics as calculated by the review authors. Directional associations were graded as convincing, highly suggestive, suggestive, or weak; and null associations were graded as suggestive or weak. More details of the methodologies used are provided in the first paper in this umbrella review series [33].

**Table 1:** Criteria for credibility assessment

Category	Associations
**Directional associations**	
Convincing	
	With statistical significance of *P* < 10^−6^
	Based on ˃1000 cases (or ˃20,000 participants for continuous outcomes)
	For which largest component study reports a statistically significant result (*P* < 0.05) and has a 95% prediction interval that excludes the null
	Which do not have large heterogeneity (*I*^2^<50%)
	Show no evidence of small study effects (*P* ˃ 0.10) or of excess significance bias (*P* ˃ 0.10)
Highly suggestive	With statistical significance of *P* < 10^−6^
	Based on ˃1000 cases (or ˃20,000 participants for continuous outcomes)
	For which largest component study reports a statistically significant result (*P* < 0.05)
Suggestive	With statistical significance of *P* < 0.01
	Based on ˃1000 cases (or ˃20,000 participants for continuous outcomes)
Weak	With statistical significance of *P* < 0.05
**Null associations**	
Suggestive	Based on ˃1000 cases (or ˃20,000 participants for continuous outcomes)
	Which do not have large heterogeneity (*I*^2^<50%)
	With statistical significance of *P* > 0.10
Weak	With statistical significance of 0.05<*P* < 0.01

## RESULTS

### Overview of search results

Of the 287 systematic reviews identified for this umbrella review series [33] (Figure S1 in the **Online Supplementary Document**), 76 reviews (33 with meta-analyses) [40–112] examined relationships between folate exposure and the risk of neuropsychiatric disorders (Table S1 in the **Online Supplementary Document**). By category of outcomes, these reviews reported on neurological disorders (epilepsy, amyotrophic lateral sclerosis, migraine, AD, PD, dementia), cognitive function, and psychiatric disorders (depression, bipolar disorder, autism spectrum disorder (ASD), attention deficit hyperactivity disorder, schizophrenia, psychosis, and obsessive-compulsive disorder). Multiple sclerosis was classified as an autoimmune condition and related findings were reported in the previous paper in this series [113].

Most of the included reviews pooled from cross-sectional or case-control studies. Of the 44 unique associations identified from the meta-analyses, only 13 (30%) were based entirely on prospective investigations (RCTs or prospective cohorts) and 11 (25%) combined findings from prospective cohort studies with those from case-control or cross-sectional studies. Plasma/serum folate concentrations were examined in 26 (59%) associations and FA supplementation in 12 (27%) associations. A total of 18 unique associations were reported only in syntheses without meta-analysis, primarily related to cognitive function and depressive disorder. Most of these syntheses included a small number (<5) of component studies (Table S2 in the **Online Supplementary Document**).

### Risk of bias assessment

**Figure 1:**
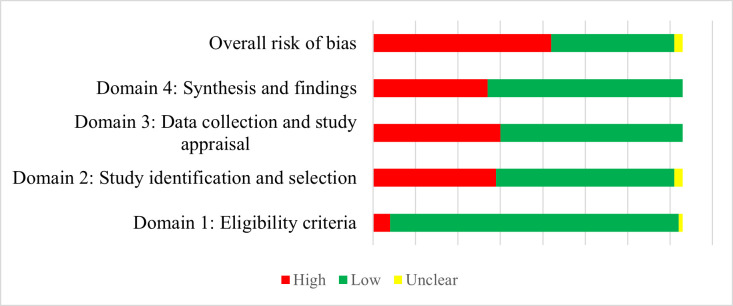
Risk of bias assessment of the included syntheses examining the relationship between folate intake/status and neuropsychiatric disorders.

More than half of the reviews (58%) included in this synthesis had a high overall risk of bias (**Figure 1**; Table S3 in the **Online Supplementary Document**). Specifically, the risk of bias was generally low among the studies in the domains of defining eligibility criteria and executing search strategies (93%), but approximately half of the reviews had a high risk of bias in the domains of identifying and selecting studies (40%), collecting data and assessment of the included studies (41%), and synthesising the findings (37%).

### Epilepsy

One meta-analysis [40] examined the relationship between FA supplementation and seizure frequency among individuals with epilepsy. The authors pooled two RCTs comparing FA dose of 15 mg/d with placebo for a duration of 3–6.5 months and reported no effect (75 participants, 18 cases; odds ratio (OR) = 0.98; 95% CI = 0.32, 2.98; *I*^2^ = 0%). The same authors qualitatively synthesised the effect of FA supplementation (5–15 mg/d for 3–6 months) compared to placebo on intelligence and behaviour in individuals with epilepsy, examining RCTs, blinded crossover studies, or blinded controlled studies. No significant difference between FA treatment and placebo groups in intelligence (three trials, 172 participants) or behaviour (three trials, 128 participants) was apparent.

### Amyotrophic lateral sclerosis 

One meta-analysis [41] pooled three case-control studies, comparing plasma folate concentration in individuals with amyotrophic lateral sclerosis and in healthy controls. No significant difference was observed between the two groups (338 participants, 169 cases; mean difference (MD) = −0.52 ng/mL; 95% CI = −1.89, 0.84); *I*^2^ = 83%).

### Migraine

One meta-analysis [42] synthesised case-control studies reporting on a relationship between serum folate concentration and migraine. Individuals with migraine had significantly lower serum folate concentration (1406 participants, 744 cases; standardised mean difference (SMD) = −0.36; 95% CI = −0.68, −0.05; *I*^2^ = 87%) compared to healthy controls. The magnitude of the association was attenuated among adults (1,215 participants, 643 cases; SMD = −0.11; 95% CI = −0.25, 0.01; *I*^2^ = 6%). Serum folate concentration was not significantly different when comparing individuals with migraine with aura with healthy controls (405 participants, 144 cases; SMD = −0.17; 95% CI = −0.44, 0.10; *I*^2^ = 47%) or with individuals with migraine without aura (292 participants, 144 cases; SMD = −0.16; 95% CI = −0.35, 0.04; *I*^2^ = 44%).

### Dementia

A higher intake of dietary folate in older individuals was associated with a reduced risk of dementia (13,473 participants; hazard ratio = 0.61; 95% CI = 0.47, 0.78; *I*^2^ = 39%) [43] examined in prospective cohorts. A lower plasma/serum folate concentration in older individuals was also associated with an increased risk of dementia (6654 participants; OR = 1.76; 95% CI = 1.24, 2.50; *I*^2^ = 81%) [43]. Older individuals with vascular dementia had significantly lower plasma folate concentrations compared to healthy controls (5,209 participants, 308 cases; SMD = −0.80; 95% CI = −1.20, −0.39; *I*^2^ = 82.6%) [44].

A narrative synthesis examined three prospective cohorts of individuals with mild cognitive impairment and if serum folate concentration was associated with their progression to all-cause dementia [45]. Two of the cohorts (n = 246) reported that a higher serum folate concentration predicted a lower risk of progression, while one (n = 55) showed a non-significant trend.

### AD

A higher total folate intake was significantly associated with a reduced risk of AD in older individuals examined in prospective cohorts (4,325 participants; risk ratio (RR) = 0.50; 95% CI = 0.25, 0.76; *I*^2^ = 32.9%) [46]. This inverse association was consistent in individuals with a higher intake (≥400 *μ*g/d) (608 participants; RR = 0.44; 95% CI = 0.18, 0.71; *I*^2^ = 35.3%; however, there was no association in those with a lower intake (<400 *μ*g/d) (426 participants; RR = 1.15; 95% CI = 0.28, 2.02; *I*^2^ = 0%) [46].

Six meta-analyses [44,46–50] reported on the relationship between plasma/serum or cerebrospinal fluid folate concentration and AD, primarily synthesising case-control and cross-sectional studies. Based on cross-sectional studies in the general population, plasma/serum folate concentration was reported to be significantly lower in individuals with AD compared to healthy controls (7,814 participants, 3,496 cases; SMD = −0.60; 95% CI = −0.65, −0.55; *I*^2^ = 97.2%) [46]. Lower folate concentration was not associated with a higher risk of AD (401 participants, 238 cases; OR = 0.86; 95% CI = 0.46, 1.26; *I*^2^ = 0%) in individuals classified as without folate deficiency (≥13.5 nmol/L); however, it was associated with a higher AD risk in those with potential folate deficiency (<13.5 nmol/L) [46].

Older individuals with AD had significantly lower plasma/serum folate concentration than healthy controls (7833 participants, 2932 cases; SMD = −0.50; 95% CI = −0.64, −0.36; *I*^2^ = 85.5%) [44] based on prospective cohorts and case-control studies. Meta-analysis of five prospective cohorts of older adults with potential folate deficiency (<13.5 nmol/L) also indicated that lower plasma folate concentration was associated with a higher risk of AD (1,835 participants; RR = 1.88; 95% CI = 1.20, 2.57; *I*^2^ = 0%) [46]. One meta-analysis of case-control studies [50] reported that individuals with AD had significantly lower cerebrospinal fluid folate concentration than healthy controls (845 participants, 307 cases; MD = −0.56; 95% CI = −0.73, −0.38).

### PD

One meta-analysis of case-control studies reported a null association (OR = 1.01; 95% CI = 0.68, 1.34; *I*^2^ = 0%) between a higher intake of dietary folate and the risk of PD [51]. The same authors reported no difference in the plasma/serum folate concentration between individuals with PD and healthy controls (1,659 participants, 735 cases; SMD = −0.12 nmol/L; 95% CI = −0.28, 0.04; *I*^2^ = 49.2%) [51]. The findings were similar in individuals treated with Levodopa (SMD = −0.14 nmol/L; 95% CI = −0.33, 0.04; total n = 1,406; case n = 692; *I*^2^ = 58.5%) and in those not treated with Levodopa (253 participants, 43 cases; SMD = 0.02 nmol/L; 95% CI = −0.31, 0.35; *I*^2^ = 0%). In non-overlapping case-control studies conducted in China, individuals with PD had a significantly lower plasma folate concentration compared to healthy controls (2,808 participants, 1,705 cases; SMD = −0.31; 95% CI = −0.47, −0.15; *I*^2^ = 74.3%) [52].

### Cognitive function

A meta-analysis of four RCTs conducted in older individuals without dementia at baseline reported that FA supplementation (0.75–15 mg/d for 1–36 months), compared to placebo, did not have an effect on cognitive function (1083 participants; SMD = 0.10; 95% CI = −0.06, 0.25) [53]. The authors aggregated and standardised findings from different cognitive tests.

A lower serum folate concentration was associated with an increased risk of cognitive impairment in older individuals (9,747 participants; OR = 1.66; 95% CI = 1.40, 1.96) [54]. The association remained significant in males (2,300 participants; OR = 1.95; 95% CI = 1.55, 2.46) and females (3,115 participants; OR = 1.90; 95% CI = 1.55, 2.32). A sensitivity analysis limited to prospective cohorts showed a comparable relationship with a reduced magnitude (3103 participants; OR = 1.40; 95% CI = 1.06, 1.84).

Several narrative syntheses [55–58] examined the relationship between dietary, total folate intake, or FA supplementation and cognitive function in various populations. The evidence on the association between dietary folate or total folate intake and risk of cognitive decline was inconclusive [55].

In individuals with cognitive impairment or mild/moderate dementia, FA supplementation significantly improved memory, information processing speed, or sensory-motor speed in three RCTs (n = 1,059) and one prospective cohort (sample size not reported) but showed no difference in another RCT (n = 7) [56].

In two RCTs with children with Down syndrome, the effect of FA supplementation on cognitive performance appeared to be dependent on the dose: improvement on the cognitive function was not significant in a study with a small dose (0.1 mg/d) but significant in a study with a larger dose (1 mg/kg/d) [57]. In Chinese older individuals with mild cognitive impairment or AD, FA supplementation (0.4–1.25 mg/d for 6–24 months) was effective in improving cognitive scores compared to placebo or conventional treatments [58].

### Depression or depressive symptoms

In individuals diagnosed with depression and taking antidepressants, consuming FA supplements (0.5–10 mg/d for 6–12 weeks), compared to taking antidepressants alone, was not effective in improving depressive symptoms (671 participants; SMD = 0.49; 95% CI = −0.31, 1.29; *I*^2^ = 93%) based on three RCTs and one open-label trial [59]. The authors noted differences in medications used, study durations, nutrient dosage, and participant characteristics as potential sources of high heterogeneity. In a meta-analysis combining prospective cohorts and cross-sectional studies examining pregnant women, taking FA supplementation, compared to none, was association with a reduced risk of perinatal depression (12,881 participants, 2,378 cases; OR = 0.71; 95% CI = 0.59, 0.83; *I*^2^ = 74%) [60]. Serum/red blood cell folate concentration was also significantly lower in women with perinatal depression (6,442 participants, 1,742 cases; SMD = −0.13; 95% CI = −0.18, −0.07; *I*^2^ = 61%) compared to healthy controls [60] examined in prospective cohorts.

Eight qualitative syntheses reported 11 unique associations. The relationship between dietary folate intake and depressive symptoms in the general population was inconclusive for male and null for female [61] based on prospective cohorts and cross-sectional studies. Pooling two prospective cohorts in Europe examining individuals without depression at baseline, Sanhueza *et al.* [62] reported that a higher intake of dietary folate was associated with a reduced risk of depressive episodes, but noted that having a history of depression may confound the pooled risk estimate. In pregnant women, the relationship between dietary folate intake and a risk of postpartum depression was null or weak based on three prospective cohorts and one cross-sectional study [63]. The association between plasma folate concentration and the risk of antenatal or postpartum depression in pregnant women was inconclusive [64].

Methylfolate supplementation was effective in reducing depressive symptoms in older individuals with depression based on two RCTs and one pre-post intervention trial [65]. In outpatients with major depressive disorder receiving antidepressants, FA supplementation did not produce difference in the treatment response based on two RCTs [66].

Among individuals with major depressive disorder taking antidepressants, those with low plasma folate concentration showed higher rates of non-response or resistance to treatments, relapses, or recurrence of depressive symptoms based on RCTs and open-label intervention trials [67].

### Bipolar disorder

One meta-analysis of case-control studies reported that individuals with bipolar disorder had significantly lower serum folate concentration (1,241 participants, 481 cases; g = −0.21; 95% CI = −0.39, −0.03; *I*^2^ = 42%) [68]. In one narrative synthesis examining individuals bipolar-I disorder with current acute mania and initiating valproate or individuals who had been taking lithium for >12 months, the two RCTs included in the synthesis showed different results regarding the effect of FA supplementation on improving symptom score [69].

### ASD

Based on cross-sectional and case-control studies, three meta-analyses [70–72] reported on the relationship between plasma/serum folate concentration and the risk of ASD. The plasma folate concentrations were not different between children with ASD and healthy controls in a meta-analysis of cross-sectional studies (1,665 participants, 710 cases; MD = 0.05; 95% CI = −1.28, 1.38; *I*^2^ = 98.3%) [72]. The finding of null association was comparable across the three meta-analyses.

Four meta-analyses examined the association between reported maternal FA supplement use and the risk of ASD in offspring [73–76]. Maternal FA use was associated with a significantly reduced risk of ASD in children aged 1.5–15 years (739,226 participants, 6,396 cases; OR = 0.58; 95% CI = 0.46, 0.75; *I*^2^ = 87%) [74], based on prospective cohort and case-control studies. The magnitude of the association was attenuated in another meta-analysis limited to prospective cohort studies that partially overlap with the analysis by Iglesias *et al.* (616,911 participants, 2,794 cases; RR = 0.90; 95% CI = 0.79, 0.998; *I*^2^ = 42.6%) [73]. This inverse association was consistently observed in subgroups of studies conducted in Asia (19,027 participants, 15,781 cases; RR = 0.67; 95% CI = 0.46, 0.97; *I*^2^ = 64.9%), Europe (596,925 participants, 2,258 cases; RR = 0.84; 95% CI = 0.68, 0.99; *I*^2^ = 53.5%), and the USA (15,953 participants, 675 cases; RR = 0.41; 95% CI = 0.17, 0.99; *I*^2^ = 68%) [73]. In younger children aged between 11 months and 6.5 years, no difference was observed between maternal FA supplementation ≥400 *μ*g/d and <400 *μ*g/d in terms of offspring motor development (8,805 participants; SMD = −0.02; 95% CI = −0.08, 0.04; *I*^2^ = 20%) or mental development (11,302 participants; SMD = −0.06; 95% CI = −0.11, 0.00; *I*^2^ = 35%) [74].

Two unique associations were additionally reported by narrative syntheses. The evidence on the relationship between dietary folate intake and the risk of ASD was inconclusive [77]. The evidence on the association between maternal plasma folate concentration and the risk of ASD in offspring was also inconclusive [78].

### Attention deficit hyperactivity disorder 

One meta-analysis of cross-sectional studies reported that children with attention deficit hyperactivity disorder (mean age ranging from 7.7 to 9.2 years) had higher plasma/serum folate concentration compared to healthy controls (1,121 participants, 537 cases; MD = 0.92; 95% CI = 0.01, 1.76; *I*^2^ = 99%) [72].

### Psychosis

A meta-analysis of six cross-sectional studies reported that the serum folate concentration was significantly lower in individuals with first-episode psychosis compared to healthy controls (827 participants, 421 cases; g = −0.62; 95% CI = −1.18, −0.07; *I*^2^ = 92%) [79].

### Schizophrenia

Based on case-control studies, serum folate concentration was significantly lower in individuals with schizophrenia than in healthy controls (2,739 participants, 1,463 cases; SMD = −0.57; 95% CI = −0.76, −0.37; *I*^2^ = 83%) [80]. In individuals diagnosed with schizophrenia and being treated with antipsychotics, FA supplementation (2–500 mg/d for 12 − 16 weeks) did not effectively improve total symptom score (121 participants; SMD = −0.25; 95% CI = −0.62, 0.11; *I*^2^ = 0%) or negative symptom score (104 participants; SMD = −0.20; 95% CI = −0.59, 0.18; *I*^2^ = 0%) in RCTs [81].

### Obsessive-compulsive disorder

Two meta-analyses compared the plasma/serum folate concentrations between individuals with obsessive-compulsive disorder and healthy controls and reported a null association (309 participants, 172 cases; SMD = −0.09; 95% CI = −0.75, 0.58; *I*^2^ = 88%) [82].

### Credibility assessment

We conducted a credibility assessment for all identified unique associations (**Table 2**). All but two associations were assessed as having weak credibility, primarily owing to small sample sizes. Seven associations were downgraded from a potentially suggestive to a weak level due to unavailable data. Two associations were assessed to be at a suggestive level of credibility: use of FA supplement, compared to none, was associated with a reduced risk of perinatal depression in pregnant women (OR = 0.71; 95% CI = 0.59, 0.83) [60]; and maternal FA supplementation, compared to none, was associated with a reduced risk of ASD in offspring (OR = 0.58; 95% CI = 0.46, 0.75) [74].

**Table 2:** Identified unique associations between folate intake/status and neuropsychiatric outcomes

Author (year)	Outcome, subgroup	Primary study design	Exposure	Total number (number of cases)	Metric	Summary estimate (95% CI)	Estimated *P*-value	*I*^2^ (%)	Credibility
Ranganathan *et al*. (2009) [40]	Seizure frequency, individuals with epilepsy	RCT	Supplement	75 (18)	OR	0.98 (0.32, 2.98)	0.971	0	Weak
Hu *et al*. (2023) [41]	ALS, general population	CC	Plasma	338 (169)	MD	−0.52 ng/mL (−1.89, 0.84)	0.455	83	Weak
Liampas *et al*. (2020) [42]	Migraine, general population	CC	Serum	1406 (744)	SMD	−0.36 (−0.68, −0.05)	0.025	87	Weak
Liampas *et al*. (2020) [42]	Migraine, adults	CC	Serum	1215 (643)	SMD	−0.11 (−0.25, 0.01)	0.097	6	Weak
Liampas *et al*. (2020) [42]	Migraine with aura, general population	CC	Serum	405 (144)	SMD	−0.17 (−0.44, 0.10)	0.217	47	Weak
Liampas *et al*. (2020) [42]	Migraine with aura *vs* without aura	CC	Serum	292 (144)	SMD	−0.16 (−0.35, 0.04)	0.107	44	Weak
Wang *et al*. (2022) [43]	Incident dementia, general population	PC	Dietary intake	13,473 (NR)	HR	0.61 (0.47, 0.78)	<0.001	39	Weak (insufficient data)
Wang *et al*. (2021) [44]	Vascular dementia, general population	PC, CC	Plasma	5209 (308)	SMD	−0.80 (−1.20, −0.39)	<0.001	82.6	Weak
Wang *et al*. (2022) [43]	Dementia, general population	PC, CS	Plasma	6654 (NR)	OR	1.76 (1.24, 2.50)	0.001	81	Weak (insufficient data)
Zhang *et al*. (2021) [46]	AD, general population	CC	Plasma/serum	7814 (3,496)	SMD	−0.60 (−0.64, −0.55)	<0.001	97.2	Weak
Zhang *et al*. (2021) [46]	AD, folate non-deficient population	CC	Plasma/serum	401 (238)	OR	0.86 (0.46, 1.26)	0.557	0	Weak
Zhang *et al*. (2021) [46]	AD, folate-deficient population	CC	Plasma/serum	885 (379)	OR	1.94 (1.02, 2.86)	0.011	0	Weak
Wilde *et al*. (2017) [50]	AD, general population	CC	CSF	845 (307)	MD	−0.56 (−0.73, −0.38)	<0.001	NR	Weak
Zhang *et al*. (2021) [46]	AD, general population	PC	Total intake	4325 (NR)	RR	0.50 (0.25, 0.76)	0.014	32.9	Weak (insufficient data)
Zhang *et al*. (2021) [46]	AD, general population, <400 ug/d	PC	Total intake	426 (NR)	RR	1.15 (0.28, 2.02)	0.781	0	Weak
Zhang *et al*. (2021) [46]	AD, general population, ≥400 ug/d	PC	Total intake	608 (NR)	RR	0.44 (0.18, 0.71)	0.019	35.3	Weak
Shen *et al*. (2015) [51]	PD, general population	CC	Dietary intake	NR (NR)	OR	1.01 (0.68, 1.34)	0.954	0	Weak (insufficient data)
Shen *et al*. (2015) [51]	PD, general population	CC	Plasma/serum	1659 (735)	SMD	−0.12 nmol/L (−0.28, 0.04)	0.141	49.2	Weak
Shen *et al*. (2015) [51]	PD, levodopa treated	CC	Plasma/serum	1406 (692)	SMD	−0.14 nmol/L (−0.33, 0.04)	0.138	58.5	Weak
Shen *et al*. (2015) [51]	PD, levodopa untreated	CC	Plasma/serum	253 (43)	SMD	0.02 nmol/L (−0.31, 0.35)	0.905	0	Weak
Dong *et al*. (2020) [52]	PD, Chinese population	CC	Plasma	2808 (1,705)	SMD	−0.31 (−0.47, −0.15)	<0.001	74.3	Weak
Wald *et al*. (2010) [53]	Cognitive function, general population	RCT	Supplement	1083 (NA)	SMD	0.10 (−0.06, 0.25)	0.206	NR	Weak
Michelakos *et al*. (2013) [54]	Cognitive impairment, older adults	PC, CS	Serum	9747 (NR)	OR	1.66 (1.40, 1.96)	<0.001	NR	Weak (insufficient data)
Michelakos *et al*. (2013) [54]	Cognitive impairment, older male	PC, CS	Serum	2300 (NR)	OR	1.90 (1.55, 2.46)	<0.001	NR	Weak (insufficient data)
Michelakos *et al*. (2013) [54]	Cognitive impairment, older female	PC, CS	Serum	3115 (NR)	OR	1.90 (1.55, 2.32)	<0.001	NR	Weak (insufficient data)
Sarris *et al*. (2016) [59]	Depression, general population	RCT, trial	Supplement	671 (NA)	SMD	0.49 (−0.31, 1.29)	0.229	93	Weak
Jin *et al*. (2022) [60]	Depression, pregnant women	PC, CS	Supplement	12,881 (2,378)	OR	0.71 (0.59, 0.83)	<0.001	74.4	Suggestive
Jin *et al*. (2022) [60]	Depression, pregnant women	PC	Serum/RBC	6442 (1,742)	SMD	−0.13 (−0.18, −0.07)	<0.001	60.9	Weak
Hsieh *et al*. (2019) [68]	Bipolar disorder, general population	CC	Serum	1241 (481)	g	−0.21 (−0.39, −0.03)	0.022	42.2	Weak
Prades *et al*. (2023) [72]	ASD, general population	CS	Plasma	1665 (710)	MD	0.05 (−1.28, 1.38)	0.941	98.3	Weak
Vazquez *et al*. (2019) [74]	ASD, mother-offspring pairs	PC, CC	Maternal supplement	739,226 (6,396)	OR	0.58 (0.46, 0.75)	<0.001	87	Suggestive
Wang *et al*. (2017) [73]	ASD, Asia	PC, CC	Maternal supplement	19,027 (1,581)	RR	0.67 (0.46, 0.97)	0.035	64.9	Weak
Wang *et al*. (2017) [73]	ASD, Europe	PC, CC	Maternal supplement	596,925 (2,258)	RR	0.84 (0.68, 0.99)	0.068	53.5	Weak
Wang *et al*. (2017) [73]	ASD, US	PC, CC	Maternal supplement	15,953 (675)	RR	0.41 (0.17, 0.99)	0.047	68	Weak
Vazquez *et al*. (2019) [74]	Motor development, mother-offspring pairs	PC	Maternal supplement	8805 (NA)	SMD	−0.02 (−0.08, 0.04)	0.513	20	Weak
Vazquez *et al*. (2019) [74]	Mental development, mother-offspring pairs	RCT, PC	Maternal supplement	11,302 (NA)	SMD	−0.06 (−0.11, 0.00)	0.032	35	Weak
Prades *et al*. (2023) [72]	ADHD, general population	CS	Plasma	1121 (537)	MD	0.92 (0.01, 1.76)	0.039	98.9	Weak
Firth *et al*. (2018) [79]	Psychosis, general population	CS	Serum	827 (421)	g	−0.63 (−1.18, −0.07)	0.026	92.4	Weak
Cao *et al*. (2016) [80]	Schizophrenia, general population	CC	Serum	2739 (1,463)	SMD	−0.57 (−0.76, −0.37)	<0.001	82.8	Weak
Sakuma *et al*. (2018) [81]	Schizophrenia total symptom score, individuals with schizophrenia	RCT	Supplement	121 (NA)	SMD	−0.25 (−0.62, 0.11)	0.179	0	Weak
Sakuma *et al*. (2018) [81]	Schizophrenia negative symptom score, individuals with schizophrenia	RCT	Supplement	104 (NA)	SMD	−0.20 (−0.59, 0.18)	0.308	0	Weak
Yan *et al*. (2022) [82]	OCD, general population	CC	Plasma	309 (172)	SMD	−0.09 (−0.75, 0.58)	0.790	87.6	Weak

AD – Alzheimer disease, ALS – amyotrophic lateral sclerosis, AS – Asperger’s syndrome, ASD – autism spectrum disorder, CC – case-control study, CR – case report, CS – cross-sectional study, CSF – cerebrospinal fluid, FA – folic acid, MA – meta-analysis, MD – mean difference, PC – prospective cohort, PD – Parkinson disease, PDD-NOS – pervasive developmental disorder – not otherwise specified, RBC – red blood cell, RC – retrospective cohort, RCT – randomised controlled trial, SMD – standardised mean difference, SR – systematic review, VD – vascular dementia

## DISCUSSION

### Summary of findings

The evidence landscape around folate and neuropsychiatric disorders is complex: while its volume appears to be growing, topic distribution remains rather skewed and largely concentrated on a few outcomes, such as AD, depression, and ASD. Variation in FA fortification and supplement practices across settings may also contribute to the heterogeneity we observed. In addition, most of the reviews synthesised observational studies of various designs, which may limit direct comparisons. We also found more narrative syntheses than meta-analyses, indicating a high level of heterogeneity among primary studies in scope and definition of outcome measures.

From a total of 74 systematic reviews, we identified 44 unique associations that were pooled quantitatively and 18 that were synthesised narratively. AD and dementia accounted for 13 associations (21%), depressive disorder for 12 associations (19%), and cognitive function and ASD for 9 associations (15%) each. Most of the identified reviews had a high overall risk of bias, particularly in the domains of data collection, assessment, and synthesis. Due to the small samples pooled, all but two unique associations were assessed to be at a weak level of credibility. Two associations at a suggestive level of credibility were inverse relationships between FA supplementation and perinatal depression and between maternal FA supplementation and ASD in offspring.

### Equity and global health in the evidence on neuropsychiatric outcomes

A large number of the reviews (40%) did not report the countries or regions in which the primary investigations were conducted. The remaining reviews were evenly divided into those that included studies from low- and middle-income countries (LMICs) (31%) and those that were limited to studies from high-income countries (HICs) (29%). We observed a temporal trend, where reviews incorporating studies conducted in LMICs started around 2015; the syntheses published earlier did not report the geographical distribution of the primary studies pooled or were limited to studies in HICs. This may reflect a growing awareness and research interest in neuropsychiatric health in LMICs in the past decade. A remaining concern, however, is that only a few LMICs contribute to the body of evidence, notably China, Turkey, and India. More research in other lower-income countries will enrich the evidence landscape and enhance our understanding on the complexity of the relationship between folate intake and various neuropsychiatric conditions.

Indeed, over 80% of the mental health conditions occur in 153 countries classified as LMICs [114,115], with depression projected to be one of the top three causes of death in LMICs by 2030 [115]. Factors related to the high prevalence of mental health problems in LMICs may include rapid urbanisation, economic distress, lack of social infrastructure and resources, and younger population demographics who report higher lifetime prevalence of mental disorders and higher number of lifetime disorders [6,116,117]. The burden of neurological disorders also appears to be the greatest in LMICs [118]. This disproportionate burden of neuropsychiatric conditions in LMICs emphasises the need for more research conducted in these countries or consideration of socioeconomic moderators.

### Biological plausibility of associations between folate and depression and ASD

5-methyltetrahydrofolate is the primary form of folate in circulation and is concentrated across the blood-brain barrier into cerebrospinal fluid and synaptic regions. It donates its methyl group to homocysteine to form methionine, which is converted to S-adenosylmethionine (SAM), the universal methyl donor for cellular methylation reactions [119,120], except in the methylation 2'-deoxyuridine-5′-monophosphate to 2'-deoxythymidine-5′-monophosphate (dTMP), where the carbon used for the methylation is derived from methylenetetrahydrofolate. Decreased methylation of neuronal membrane phospholipids may affect neurotransmitter function [119].

The effect of low or deficient folate on the nervous system has been well demonstrated in the prevention of primary and recurrent neural tube defects by FA supplementation [121,122]; the neuropsychiatric complications of antifolate drugs [123–125]; and the disorders of peripheral nerve and spinal cord functions [126,127]. With regard to folate in depression, two hypotheses of aetiological pathways have been proposed: cellular methylation deficit and hydroxylation of tyrosine and tryptophan [120].

First, methylfolate seems to be effective in folate treatment of cases with major depressive disorder [65], while FA is not [67]. Furthermore, an association with the ineffectiveness of antidepressant treatments was observed in patients with low blood folate levels [67]. The ability to restore optimal blood folate levels is different between methylfolate and FA [128] where the former is much more effective than the latter. The effective bioavailability of folate from FA treatment is, in fact, severely limited by the low saturation level of the dihydrofolate reductase enzyme, which is necessary for the conversion of FA to tetrahydrofolate and then to methylfolate. Methylfolate supplementation, however, does not suffer from this limitation, as methylfolate is directly bioavailable. It might be useful to highlight this difference in clinical practice using these two supplements.

There are also differences between methylfolate and SAM treatments, with the latter, in addition to being a methyl group donor, also acting as a potent inhibitor of the methylenetetrahydrofolate reductase (MTHFR) enzyme, which is responsible for methylfolate synthesis. A negative feedback loop is therefore generated between MTHFR and SAM. Treatment with SAM, on the one hand, temporarily increases the availability of methyl groups for methylation reactions, but on the other, reduces the methylfolate synthesis reaction performed by MTHFR. This causes the MTHFR substrate, methylene- tetrahydrofolate, to be diverted to the synthesis of 2'-deoxythymidine-5′-monophosphate performed by thymidylate synthase (TS). The effect of SAM treatment could, at least in part, be due to improved genomic stability.

Low folate status may also be related to depression through hydroxylation of the amino acids tyrosine and tryptophan in the synthesis of catecholamines and 5-hydroxytryptamine [120]. Dihydrofolate reductase, along with dihydropteridine reductase, is involved in the regeneration of tetrahydropterin, which are essential for the hydroxylation in the brain [129]. Pteridine is required for tryptophan hydroxylase, the rate-limiting enzyme in the synthesis of serotonin, and for tyrosine hydroxylase in the synthesis of dopamine [120].

Cerebral folate deficiency has been suggested as a potential risk associated with ASD [130,131], as well as various neurological deficits [132] and neuropsychiatric conditions [133,134]. Intake of dietary folate appears to vary substantially among individuals with ASD, with more studies reporting inadequate intake often accompanied by high food selectivity and eating problems [135,136]. In addition, folate receptor alpha antibodies, which disrupt the transport of 5-methyltetrahydrofolate across the blood-brain barrier and consequently contributing to cerebral folate deficiency [137], have been observed in individuals with ASD [138]. A recent systematic review [138] reported 38% pooled prevalence of cerebral folate deficiency in individuals with ASD. Some genetic factors, such as MTHFR polymorphisms, also appears to play an important role on the risk of ASD [83,139].

Maternal folate status during pregnancy has been widely examined in relation to the risk of offspring ASD because periconceptional period is a critical window for foetal neurogenesis [140], for which high concentrations of methyl donors are required [141,142]. Most studies, however, are limited by incomplete or heterogenous measurements of folate intake/status during the perinatal period, *e.g.* folate intake was self-reported, or biomarkers were measured at different trimesters [23], as well as insufficient examination of other potential risk factors, *e.g.* maternal obesity and pre-gestational or gestational diabetes [31,143].

### Limitations

This review has several limitations. First, most of the systematic reviews in our syntheses pooled primarily from observational studies, particularly cross-sectional studies and case-control studies, which cannot inform a causal relationship between folate exposure and specific outcomes. Most of the unique associations examined the differences in plasma folate concentrations between cases and controls. Second, sex-stratified analyses were not available except for one unique association (serum folate concentration and cognitive impairment among older adults). Sex differences in the prevalence, age of onset, and clinical manifestations of neuropsychiatric disorders have been well-established [144–147]. Some reports also point to sex differences in cognitive function, such as learning and memory [2,148,149]. Third, study population was not adequately described in many reviews. Underlying morbidities, duration of illness, and use of medications may be important moderators in the relationship between folate exposure and neuropsychiatric disorders. Lastly, interactions with other nutrients involved in one-carbon metabolism, *e.g.* vitamins B_12_ and B_2_, and choline, were not examined in this review. Future research on the role of the interactions among these nutrients will provide further insights on the folate – neuropsychiatric health relationship in a more comprehensive context.

## CONCLUSIONS

In our review of the available evidence on the relationship between folate exposure and neuropsychiatric disorders, we identified 62 unique associations (44 from meta-analyses) from 74 systematic reviews across 14 outcomes. The majority of the included reviews included only observational studies and had high risk of bias. We identified two associations assessed to be at a suggestive level of credibility: FA supplementation was associated with a reduced risk of perinatal depression and maternal FA supplementation was associated with a reduced risk of ASD in offspring. These findings, along with most of the findings we reported here, should be further examined in well-designed RCTs to adequately inform policy decisions or population-level interventions. Sex-stratified analyses, which are important considerations in neuropsychiatric research, were mostly unavailable. More prospective intervention studies designed for dose-response analyses as well as dose-response analyses at the meta-analysis level using individual patient data will add to the current knowledge on folate and neuropsychiatric disorders and better inform clinical and policy decision makers.

## Additional material


Online Supplementary Document

## Data Availability

**Data availability:** The data supporting the findings of this study are available within the article and its supplementary materials.

## References

[1] Huang Y Li Y Pan H Han L Global, regional, and national burden of neurological disorders in 204 countries and territories worldwide. J Glob Health. 2023;13:04160. 10.7189/jogh.13.04160 38018250 PMC10685084

[2] Feigin VL Vos T Nichols E Owolabi MO Carroll WM Dichgans M The global burden of neurological disorders: translating evidence into policy. Lancet Neurol. 2020;19:255–65. 10.1016/S1474-4422(19)30411-9 31813850 PMC9945815

[3] Roth GA Mensah GA Johnson CO Addolorato G Ammirati E Baddour LM Global burden of cardiovascular diseases and risk factors, 1990–2019: Update from the GBD 2019 Study. J Am Coll Cardiol. 2020;76:2982–3021. 10.1016/j.jacc.2020.11.010 33309175 PMC7755038

[4] Galea S Uddin M Koenen K The urban environment and mental disorders: Epigenetic links. Epigenetics. 2011;6:400–4. 10.4161/epi.6.4.14944 21343702 PMC3230535

[5] Lambert KG Nelson RJ Jovanovic T Cerdá M Brains in the city: Neurobiological effects of urbanization. Neurosci Biobehav Rev. 2015;58:107–22. 10.1016/j.neubiorev.2015.04.007 25936504 PMC4774049

[6] Schumann G Benegal V Yu C Tao S Jernigan T Heinz A Precision medicine and global mental health. Lancet Glob Health. 2019;7:e32. 10.1016/S2214-109X(18)30406-6 30554758 PMC7313318

[7] GBD 2019 Mental Disorders Collaborators Global, regional, and national burden of 12 mental disorders in 204 countries and territories, 1990–2019: a systematic analysis for the Global Burden of Disease Study 2019. Lancet. 2022;396:137–50. 10.1016/s2215-0366(21)00395-3 35026139 PMC8776563

[8] Mao QS Guo YX Tian XL Zhao HL Kong YZ Global burden of mental disorders in 204 countries and territories results from the Global Burden of Disease Study 2021. World J Psychiatry. 2025;15:106887. 10.5498/wjp.v15.i8.106887 40837810 PMC12362664

[9] Ma K Yu J Shao W Xu X Zhang Z Zhang D Functional overlaps exist in neurological and psychiatric Disorders: A proof from brain network analysis. Neuroscience. 2020;425:39–48. 10.1016/j.neuroscience.2019.11.018 31794696

[10] Sinanović O Psychiatric disorders in neurology. Psychiatr Danub. 2012;24:S331–5. 23114812

[11] Silberstein SD Shared mechanisms and comorbidities in neurologic and psychiatric disorders. Headache. 2001;41:S11–7. 10.1046/j.1526-4610.2001.01154-3.x 11903535

[12] Taslim S Shadmani S Saleem AR Kumar A Brahma F Blank N Neuropsychiatric disorders: Bridging the gap between neurology and psychiatry. Cureus. 2024;16:e51655. 10.7759/cureus.51655 38313968 PMC10838116

[13] Smeland OB Kutrolli G Bahrami S Fominykh V Parker N Fuhrer J A genome-wide analysis of the shared genetic risk architecture of complex neurological and psychiatric disorders. Nat Neurosci. 2025;28:2439–50. 10.1038/s41593-025-02090-2 41219506

[14] Stein DJ Benjet C Gureje O Lund C Scott KM Poznyak V Integrating mental health with other non-communicable diseases. BMJ. 2019;364:l295. 10.1136/bmj.l295 30692081 PMC6348425

[15] De Hert M Correll CU Bobes J Cetkovich-Bakmas M Cohen D Asai I Physical illness in patients with severe mental disorders. I. Prevalence, impact of medications and disparities in health care. World Psychiatry. 2011;10:52–77. 10.1002/j.2051-5545.2011.tb00014.x 21379357 PMC3048500

[16] Bloom D, Cafiero E, Jane-Llopis E, Abrahams-Gessel S, Bloom L, Fathima S, *et al*. The global economic burden of non-communicable diseases: A report by the World Economic Forum and the Harvard School of Public Health. Geneva, Switzerland: World Economic Forum; 2011. Available: https://www3.weforum.org/docs/WEF_Harvard_HE_GlobalEconomicBurdenNonCommunicableDiseases_2011.pdf. Accessed: 6 August 2026.

[17] Halbreich U Schulze T Botbol M Javed A Kallivayalil RA Ghuloum S Partnerships for interdisciplinary collaborative global well-being. Asia Pac Psychiatry. 2019;11:e12366. 10.1111/appy.12366 31199084

[18] Kumagaya DY Acute electroconvulsive therapy in the elderly with schizophrenia and schizoaffective disorder: A case series. Asia Pac Psychiatry. 2019;11:e12361. 10.1111/appy.12361 31106956

[19] Frankenburg FR The role of one-carbon metabolism in schizophrenia and depression. Harv Rev Psychiatry. 2007;15:146–60. 10.1080/10673220701551136 17687709

[20] Muntjewerff JW Kahn R Blom H den Heijer M Homocysteine, methylenetetrahydrofolate reductase and risk of schizophrenia: a meta-analysis. Mol Psychiatry. 2006;11:143–9. 10.1038/sj.mp.4001746 16172608

[21] Sugden C One-carbon metabolism in psychiatric illness. Nutr Res Rev. 2006;19:117–36. 10.1079/NRR2006119 19079880

[22] Bottiglieri T Homocysteine and folate metabolism in depression. Prog Neuropsychopharmacol Biol Psychiatry. 2005;29:1103–12. 10.1016/j.pnpbp.2005.06.021 16109454

[23] Bender A Hagan KE Kingston N The association of folate and depression: A meta-analysis. J Psychiatr Res. 2017;95:9–18. 10.1016/j.jpsychires.2017.07.019 28759846

[24] Tsuchimine S Saito M Kaneko S Yasui-Furukori N Decreased serum levels of polyunsaturated fatty acids and folate, but not brain-derived neurotrophic factor, in childhood and adolescent females with depression. Psychiatry Res. 2015;225:187–90. 10.1016/j.psychres.2014.11.018 25466229

[25] García-Miss MR Perez-Mutul J Lopez-Canul B Folate, homocysteine, interleukin-6, and tumor necrosis factor levels, but not the methylenetetrahydrofolate reductase C677T polymorphism, are risk factors for schizophrenia. J Psychiatry Res. 2010;44:441–6. 10.1016/j.jpsychires.2009.10.011 19939410

[26] Wang D Zhai J Liu D Serum folate levels in schizophrenia: a meta-analysis. Psychiatry Res. 2016;235:83–9. 10.1016/j.psychres.2015.11.045 26652840

[27] Zheng W Li W Qi H Xiao L Sim K Ungvari GS Adjunctive folate for major mental disorders: A systematic review. J Affect Disord. 2020;267:123–30. 10.1016/j.jad.2020.01.096 32063563

[28] Taylor MJ Carney SM Goodwin GM Geddes JR Folate for depressive disorders: Systematic review and meta-analysis of randomized controlled trials. J Psychopharmacol. 2004;18:251–6. 10.1177/0269881104042630 15260915

[29] Dartois LL Stutzman DL Morrow M L-methylfolate augmentation to antidepressants for adolescents with treatment-resistant depression: A case series. J Child Adolesc Psychopharmacol. 2019;29:386–91. 10.1089/cap.2019.0006 31058543

[30] Freeman MP Savella GM Church TR Góez-Mogollón L Sosinsky AZ Noe OB A prenatal supplement with methylfolate for the treatment and prevention of depression in women trying to conceive and during pregnancy. Ann Clin Psychiatry. 2019;31:4–16. 10.1177/104012371903100107 30699214

[31] Firth J Stubbs B Sarris J Rosenbaum S Teasdale S Berk M The effects of vitamin and mineral supplementation on symptoms of schizophrenia: a systematic review and meta-analysis. Psychol Med. 2017;47:1515–27. 10.1017/S0033291717000022 28202095

[32] Bo Y Zhu Y Tao Y Li X Zhai D Bu Y Association Between Folate and Health Outcomes: An Umbrella Review of Meta-Analyses. Front Public Health. 2020;8:550753. 10.3389/fpubh.2020.550753 33384976 PMC7770110

[33] Yoo S Montazeri A Bennett D Bo Y Chen P Duthie S Folate and global health umbrella review series, part 1: methodological framework and syntheses on anaemia and neural tube defects. J Glob Health. 2026;16:04014. 10.7189/jogh.16.04014 41612855 PMC12856383

[34] Gianfredi V Nucci D Amerio A Signorelli C Odone A Dinu M What can we expect from an umbrella review? Adv Nutr. 2022;13:684–5. 10.1093/advances/nmab150 37270205 PMC8970837

[35] Belbasis L Bellou V Ioannidis JPA Conducting umbrella reviews. BMJ Med. 2022;1:e000071. 10.1136/bmjmed-2021-000071 36936579 PMC9951359

[36] Whiting P Savovic J Higgins J Caldwell D Reeves B Shea B ROBIS: a new tool to assess risk of bias in systematic reviews was developed. J Clin Epidemiol. 2016;69:225–34. 10.1016/j.jclinepi.2015.06.005 26092286 PMC4687950

[37] Borenstein M, Hedges LV, Higgins JPT, Rothstein HR. Prediction intervals. In: Borenstein M, Hedges LV, Higgins JPT, Rothstein HR, editors. Introduction to Meta-Analysis. Second edition. Hoboken, New Jersey, USA: John Wiley & Sons, Inc.; 2021. p. 119–125.

[38] Egger M Davey Smith G Minder C Bias in meta-analysis detected by a simple, graphical test. BMJ. 1997;315:629–34. 10.1136/bmj.315.7109.629 9310563 PMC2127453

[39] Ioannidis JP Trikalinos TA An exploratory test for an excess of significant findings. Clin Trials. 2007;4:245–53. 10.1177/1740774507079441 17715249

[40] Ranganathan LN Ramaratnam S Vitamins for epilepsy. Cochrane Database Syst Rev. 2005;(2):CD004304. 15846704 10.1002/14651858.CD004304.pub2

[41] Hu N Wang X The level of homocysteine in amyotrophic lateral sclerosis: a systematic review and meta-analysis. Neurol Sci. 2023;44:1185–92. 10.1007/s10072-022-06518-6 36422727

[42] Liampas I Siokas V Mentis AFA Aloizou AM Dastamani M Tsouris Z Serum homocysteine, pyridoxine, folate, and vitamin B12 levels in migraine: Systematic review and meta-analysis. Headache. 2020;60:1508–34. 10.1111/head.13892 32615014

[43] Wang Z Zhu W Xing Y Jia J Tang Y B vitamins and prevention of cognitive decline and incident dementia: A systematic review and meta-analysis. Nutr Rev. 2022;80:931–49. 10.1093/nutrit/nuab057 34432056

[44] Wang Q Zhao J Chang H Liu X Zhu R Homocysteine and folic acid: Risk factors for Alzheimer’s disease—An updated meta-analysis. Front Aging Neurosci. 2021;13:665114. 10.3389/fnagi.2021.665114 34122042 PMC8188894

[45] Cooper C Sommerlad A Lyketsos CG Livingston G Modifiable predictors of dementia in mild cognitive impairment: A systematic review and meta-analysis. Am J Psychiatry. 2015;172:323–34. 10.1176/appi.ajp.2014.14070878 25698435

[46] Zhang X Bao G Liu D Yang Y Li X Cai G The association between folate and Alzheimer’s disease: A systematic review and meta-analysis. Front Neurosci. 2021;15:661198. 10.3389/fnins.2021.661198 33935641 PMC8079632

[47] Van Dam F Van Gool WA Hyperhomocysteinemia and Alzheimer’s disease: A systematic review. Arch Gerontol Geriatr. 2009;48:425–30. 10.1016/j.archger.2008.03.009 18479766

[48] Lopes da Silva S Vellas B Elemans S Luchsinger J Kamphuis P Yaffe K Plasma nutrient status of patients with Alzheimer’s disease: Systematic review and meta-analysis. Alzheimers Dement. 2014;10:485–502. 10.1016/j.jalz.2013.05.1771 24144963

[49] Shen L Ji HF Associations between homocysteine, folic acid, vitamin B12 and Alzheimer’s disease: Insights from meta-analyses. J Alzheimers Dis. 2015;46:777–90. 10.3233/JAD-150140 25854931

[50] de Wilde MC Vellas B Girault E Yavuz AC Sijben JW Lower brain and blood nutrient status in Alzhiemer’s disease: Results from meta-analyses. Alzheimers Dement (N Y). 2017;3:416–31. 10.1016/j.trci.2017.06.002 29067348 PMC5651428

[51] Shen L Associations between B vitamins and Parkinson’s disease. Nutrients. 2015;7:7197–208. 10.3390/nu7095333 26343714 PMC4586528

[52] Dong B Wu R Plasma homocysteine, folate and vitamin B12 levels in Parkinson’s disease in China: A meta-analysis. Clin Neurol Neurosurg. 2020;188:105587. 10.1016/j.clineuro.2019.105587 31733593

[53] Wald DS Kasturiratne A Simmonds M Effect of Folic Acid, with or without Other B Vitamins, on Cognitive Decline: Meta-Analysis of Randomized Trials. Am J Med. 2010;123:527.e2. 10.1016/j.amjmed.2010.01.017 20569758

[54] Michelakos T Kousoulis AA Katsiardanis K Dessypris N Anastasiou A Katsiardani KP Serum folate and B12 levels in association with cognitive impairment among seniors: Results from the VELESTINO study in Greece and meta-analysis. J Aging Health. 2013;25:589–616. 10.1177/0898264313482488 23569157

[55] Schneider JA Tangney CC Morris MC Folic acid and cognition in older persons. Expert Opin Drug Saf. 2006;5:511–22. 10.1517/14740338.5.4.511 16774490

[56] Vogel T Dali-Youcef N Kaltenbach G Andrès E Homocysteine, vitamin B12, folate and cognitive functions: A systematic and critical review of the literature. Int J Clin Pract. 2009;63:1061–7. 10.1111/j.1742-1241.2009.02026.x 19570123

[57] Lohner S Fekete K Berti C Hermoso M Cetin I Koletzko B Effect of folate supplementation on folate status and health outcomes in infants, children and adolescents: A systematic review. Int J Food Sci Nutr. 2012;63:1014–20. 10.3109/09637486.2012.683779 22574624

[58] Gil Martínez V Salas AA Ballestín SS Vitamin Supplementation and Dementia: A Systematic Review. Nutrients. 2022;14:1–23. 35268010 10.3390/nu14051033 PMC8912288

[59] Sarris J Murphy J Mischoulon D Papakostas GI Fava M Berk M Adjunctive nutraceuticals for depression: A systematic review and meta-analyses. Am J Psychiatry. 2016;173:575–87. 10.1176/appi.ajp.2016.15091228 27113121

[60] Jin X Cheng Z Yu X Tao Q Huang R Wang S Continuous supplementation of folic acid in pregnancy and the risk of perinatal depression–A meta-analysis. J Affect Disord. 2022;302:258–72. 10.1016/j.jad.2022.01.080 35066009

[61] Murakami K Sasaki S Dietary intake and depressive symptoms: A systematic review of observational studies. Mol Nutr Food Res. 2010;54:471–88. 10.1002/mnfr.200900157 19998381

[62] Sanhueza C Ryan L Foxcroft DR Diet and the risk of unipolar depression in adults: Systematic review of cohort studies. J Hum Nutr Diet. 2013;26:56–70. 10.1111/j.1365-277X.2012.01283.x 23078460

[63] Sparling TM Henschke N Nesbitt RC Gabrysch S The role of diet and nutritional supplementation in perinatal depression: a systematic review. Matern Child Nutr. 2017;13:e12235. 10.1111/mcn.12235 26840379 PMC6865932

[64] Trujillo J Vieira MC Lepsch J Rebelo F Poston L Pasupathy D A systematic review of the associations between maternal nutritional biomarkers and depression and/or anxiety during pregnancy and postpartum. J Affect Disord. 2018;232:185–203. 10.1016/j.jad.2018.02.004 29494902

[65] Frazer CJ Christensen H Griffiths KM Effectiveness of treatments for depression in older people. Med J Aust. 2005;182:627–32. 10.5694/j.1326-5377.2005.tb06849.x 15963019

[66] van der Burg KP Cribb L Firth J Karmacoska D Sarris J Nutrient and genetic biomarkers of nutraceutical treatment response in mood and psychotic disorders: a systematic review. Nutr Neurosci. 2021;24:279–95. 10.1080/1028415X.2019.1625222 31397223

[67] Morris DW Trivedi MH Rush AJ Folate and unipolar depression. J Altern Complement Med. 2008;14:277–85. 10.1089/acm.2007.0663 18370582

[68] Hsieh YC Chou LS Lin CH Wu HC Li DJ Tseng PT Serum folate levels in bipolar disorder: A systematic review and meta-analysis. BMC Psychiatry. 2019;19:305. 10.1186/s12888-019-2269-2 31640634 PMC6805488

[69] Sarris J Mischoulon D Schweitzer I Adjunctive nutraceuticals with standard pharmacotherapies in bipolar disorder: A systematic review of clinical trials. Bipolar Disord. 2011;13:454–65. 10.1111/j.1399-5618.2011.00945.x 22017215

[70] Frustaci A Neri M Cesario A Adams JB Domenici E Dalla Bernardina B Oxidative stress-related biomarkers in autism: Systematic review and meta-analyses. Free Radic Biol Med. 2012;52:2128–41. 10.1016/j.freeradbiomed.2012.03.011 22542447

[71] Gallardo-Carrasco MC Jiménez-Barbero JA Bravo-Pastor M del M Martin-Castillo D Sánchez-Muñoz M Serum vitamin D, folate and fatty acid levels in children with Autism Spectrum Disorders: A systematic review and meta-Analysis. J Autism Dev Disord. 2022;52:4708–21. 10.1007/s10803-021-05335-8 34734376 PMC9556366

[72] Prades N Varela E Flamarique I Deulofeu R Baeza I Water-soluble vitamin insufficiency, deficiency and supplementation in children and adolescents with a psychiatric disorder: a systematic review and meta-analysis. Nutr Neurosci. 2023;26:85–107. 10.1080/1028415X.2021.2020402 35034564

[73] Wang M Li K Zhao D Li L The association between maternal use of folic acid supplements during pregnancy and risk of autism spectrum disorders in children: A meta-analysis. Mol Autism. 2017;8:51. 10.1186/s13229-017-0170-8 29026508 PMC5625821

[74] Iglesias Vázquez L Canals J Arija V Review and meta-analysis found that prenatal folic acid was associated with a 58% reduction in autism but had no effect on mental and motor development. Acta Paediatr. 2019;108:600–10. 10.1111/apa.14657 30466185

[75] Guo BQ Li H Bin, Zhai DS, Ding S Bin. Association of maternal prenatal folic acid intake with subsequent risk of autism spectrum disorder in children: A systematic review and meta-analysis. Prog Neuropsychopharmacol Biol Psychiatry. 2019;94:109650. 10.1016/j.pnpbp.2019.109650 31085214

[76] Liu X Zou M Sun C Wu L Chen WX Prenatal folic acid supplements and offspring’s Autism Spectrum Disorder: A meta-analysis and meta-regression. J Autism Dev Disord. 2022;52:522–39. 10.1007/s10803-021-04951-8 33743119 PMC8813730

[77] Castro K Klein L da S Baronio D Gottfried C Riesgo R Perry IS Folic acid and autism: What do we know? Nutr Neurosci. 2016;19:310–7. 10.1179/1476830514Y.0000000142 25087906

[78] Zhong C Tessing J Lee BK Lyall K Maternal dietary factors and the risk of Autism Spectrum Disorders: A systematic review of existing evidence. Autism Res. 2020;13:1634–58. 10.1002/aur.2402 33015977 PMC9234972

[79] Firth J Carney R Stubbs B Teasdale SB Vancampfort D Ward PB Nutritional deficiencies and clinical correlates in first-episode psychosis: A systematic review and meta-analysis. Schizophr Bull. 2018;44:1275–92. 10.1093/schbul/sbx162 29206972 PMC6192507

[80] Cao B Wang DF Xu MY Liu YQ Yan LL Wang JY Lower folate levels in schizophrenia: A meta-analysis. Psychiatry Res. 2016;245:1–7. 10.1016/j.psychres.2016.03.003 27521746

[81] Sakuma K Matsunaga S Nomura I Okuya M Kishi T Iwata N Folic acid/methylfolate for the treatment of psychopathology in schizophrenia: a systematic review and meta-analysis. Psychopharmacology (Berl). 2018;235:2303–14. 10.1007/s00213-018-4926-4 29785555

[82] Yan S Liu H Yu Y Han N Du W Changes of serum homocysteine and vitamin B12, but not folate are correlated with Obsessive-Compulsive Disorder: A systematic review and meta-analysis of case-control studies. Front Psychiatry. 2022;13:754165. 10.3389/fpsyt.2022.754165 35615448 PMC9124900

[83] Main PAE Angley MT Thomas P O’Doherty CE Fenech M Folate and methionine metabolism in autism: A systematic review. Am J Clin Nutr. 2010;91:1598–620. 10.3945/ajcn.2009.29002 20410097

[84] Ellinson M Thomas J Patterson A A critical evaluation of the relationship between serum vitamin B_12_, folate, total homocysteine with cognitive impairment in the elderly. J Hum Nutr Diet. 2004;17:371–83. 10.1111/j.1365-277X.2004.00532.x 15250847

[85] Balk EM Raman G Tatsioni A Chung M Lau J Rosenberg I Vitamin B_6_, B_12_, and folic acid supplementation and cognitive function. Arch Intern Med. 2007;167:21–30. 10.1001/archinte.167.1.21 17210874

[86] Raman G Tatsioni A Chung M Rosenberg IH Lau J Lichtenstein AH Heterogeneity and lack of good quality studies limit association between folate, vitamins B-6 and B-12, and cognitive function. J Nutr. 2007;137:1789–94. 10.1093/jn/137.7.1789 17585032

[87] Jia X McNeill G Avenell A Does taking vitamin, mineral and fatty acid supplements prevent cognitive decline? A systematic review of randomized controlled trials. J Hum Nutr Diet. 2008;21:317–36. 10.1111/j.1365-277X.2008.00887.x 18721399

[88] Purnell C Gao S Callahan CM Hendrie HC Cardiovascular risk factors and incident Alzheimer disease: A systematic review of the literature. Alzheimer Dis Assoc Disord. 2009;23:1–10. 10.1097/WAD.0b013e318187541c 18703981 PMC3689425

[89] Perez L Heim L Sherzai A Jaceldo-Siegl K Sherzai A Nutrition and vascular dementia. J Nutr Health Aging. 2012;16:319–24. 10.1007/s12603-012-0042-z 22499449 PMC12878060

[90] Shah R The role of nutrition and diet in Alzheimer’s disease: A systematic review. JAMDA. 2013;14:398–402. 23419980 10.1016/j.jamda.2013.01.014

[91] Barnes JL Tian M Edens NK Morris MC Consideration of nutrient levels in studies of cognitive decline. Nutr Rev. 2014;72:707–19. 10.1111/nure.12144 25323849

[92] Colapinto CK O’Connor DL Sampson M Williams B Tremblay MS Systematic review of adverse health outcomes associated with high serum or red blood cell folate concentrations. J Public Health (Oxf). 2016;38:e84–97. 10.1093/pubmed/fdv087 26160024 PMC4894484

[93] Xie Y Feng H Peng S Xiao J Zhang J Association of plasma homocysteine, vitamin B12 and folate levels with cognitive function in Parkinson’s disease: A meta-analysis. Neurosci Lett. 2017;636:190–5. 10.1016/j.neulet.2016.11.007 27840145

[94] Murray LK Jadavji NM The role of one-carbon metabolism and homocysteine in Parkinson’s disease. Nutr Res Rev. 2019;32:218–30. 10.1017/S0954422419000106 31303188

[95] Vlachos GS Scarmeas N Dietary interventions in mild cognitive impairment and dementia. Dialogues Clin Neurosci. 2019;21:69–82. 10.31887/DCNS.2019.21.1/nscarmeas 31607782 PMC6780358

[96] Widyadharma IPE Tedyanto EH Laksmidewi AAAP Adnyana MO Samatra DPGP Folic acid supplementation improves cognitive function: A systematic review. Rom J Neurol. 2020;19:219–23. 10.37897/RJN.2020.4.1

[97] Asadi-Pooya AA Simani L Clinical trials of vitamin-mineral supplementations in people with epilepsy: A systematic review. Clin Nutr. 2021;40:3045–51. 10.1016/j.clnu.2020.10.045 33168326

[98] Huang L Zhao J Chen Y Ma F Huang G Li W Baseline folic acid status affects the effectiveness of folic acid supplements in cognitively relevant outcomes in older adults: A systematic review. Aging Ment Health. 2022;26:457–63. 10.1080/13607863.2021.1875194 33463361

[99] Harbottle L Schonfelder N Nutrition and depression: A review of the evidence. Journal of Mental Health. 2008;17:576–87. 10.1080/09638230701677746

[100] Taylor MJ Carney SM Geddes J Goodwin G Folate for depressive disorders [Review]. Cochrane Database Syst Rev. 2003;2003:CD003390. 12804463 10.1002/14651858.CD003390 PMC6991158

[101] Nahas R Sheikh O Complementary and alternative medicine for the treatment of major depressive disorder. Can Fam Physician. 2011;57:659–63. 21673208 PMC3114664

[102] Dhingra S Parle M Herbal remedies and nutritional supplements in the treatment of depression: A review. Klinik Psikofarmakol Bülteni. 2012;22:286–92. 10.5455/bcp.20120729090446

[103] Chia SC Henry J Mok YM Honer WG Sim K Fatty acid and vitamin interventions in adults with schizophrenia: A systematic review of the current evidence. J Neural Transm (Vienna). 2015;122:1721–32. 10.1007/s00702-015-1451-z 26354100

[104] Bender A Hagan K Kingston N The association of folate and depression: A meta-analysis. J Psychiatr Res. 2017;95:9–18. 10.1016/j.jpsychires.2017.07.019 28759846

[105] Firth J Stubbs B Sarris J Rosenbaum S Teasdale S Berk M The effects of vitamin and mineral supplementation on symptoms of schizophrenia: A systematic review and meta-analysis. Psychol Med. 2017;47:1515–27. 10.1017/S0033291717000022 28202095

[106] Schefft C Kilarski LL Bschor T Kohler S Efficacy of adding nutritional supplements in unipolar depression: A systematic review and meta-analysis. Eur Neuropsychopharmacol. 2017;27:1090–109. 10.1016/j.euroneuro.2017.07.004 28988944

[107] Trincado J Caneo C Is augmentation with folate effective for major depressive disorder? Medwave. 2018;18:e7156. 10.5867/medwave.2018.01.7155 29499033

[108] Balandeh E Karimian M Behjati M Mohammadi AH Serum vitamins and homocysteine levels in obsessive-compulsive disorder: A systematic review and meta-analysis. Neuropsychobiology. 2021;80:502–15. 10.1159/000514075 33744893

[109] Hoxha B Hoxha M Domi E Gervasoni J Persichilli S Malaj V Folic acid and autism: A systematic review of the current state of knowledge. Cells. 2021;10:1976. 10.3390/cells10081976 34440744 PMC8394938

[110] Sampaio AC de Matos Neto FF de Lucena Lopes L Marques IMM Tavares RM de Macedo Fernandes MV Association of the maternal folic acid supplementation with the autism spectrum disorder: A systematic review. Rev Bras Ginecol Obstet. 2021;43:775–81. 10.1055/s-0041-1736298 34784634 PMC10183865

[111] Borges-Vieira J Souza Cardoso CK Efficacy of B-vitamins and vitamin D therapy in improving depressive and anxiety disorders: A systematic review of randomized controlled trials. Nutr Neurosci. 2023;26:187–207. 10.1080/1028415X.2022.2031494 35156551

[112] Gabriel FC Oliveira M Martella BDM Berk M Brietzke E Jacka FN Nutrition and bipolar disorder: A systematic review. Nutr Neurosci. 2023;26:637–51. 10.1080/1028415X.2022.2077031 35608150

[113] Yoo S Montazeri A Bennett D Bo Y Chen P Duthie S Folate and global health review series, part 4: syntheses on folate and autoimmune diseases and skeletal outcomes. J Glob Health. 2026;16:04257. 10.7189/jogh.16.04257 42396914 PMC13329937

[114] Alloh FT Regmi P Onche I van Teijlingen E Trenoweth S Mental health in low-and middle income countries (LMICs): Going beyond the need for funding. Health Prospect. 2018;17:12–7. 10.3126/hprospect.v17i1.20351

[115] Rathod S Pinninti N Irfan M Gorczynski P Rathod P Gega L Mental Health Service Provision in Low- and Middle-Income Countries. Health Serv Insights. 2017;10:1178632917694350. 10.1177/1178632917694350 28469456 PMC5398308

[116] Javed A Lee C Zakaria H Buenaventura RD Cetkovich-Bakmas M Duailibi K Reducing the stigma of mental health disorders with a focus on low- and middle-income countries. Asian J Psychiatr. 2021;58:102601. 10.1016/j.ajp.2021.102601 33611083

[117] Cía AH Stagnaro JC Aguilar Gaxiola S Vommaro H Loera G Medina-Mora ME Lifetime prevalence and age-of-onset of mental disorders in adults from the Argentinean Study of Mental Health Epidemiology. Soc Psychiatry Psychiatr Epidemiol. 2018;53:341–50. 10.1007/s00127-018-1492-3 29459988

[118] Feigin VL Vos T Global burden of neurological disorders: From global burden of disease estimates to actions. Neuroepidemiology. 2019;52:1–2. 10.1159/000495197 30472717

[119] Reynolds EH Carney MW Toone BK Methylation and mood. Lancet. 1984;324:196–8. 10.1016/S0140-6736(84)90482-3 6146753

[120] Abou-Saleh MT Coppens A The biology of folate in depression: Implications for nutritional hypotheses of the psychoses. J Psychiatr Res. 1986;20:91–101. 10.1016/0022-3956(86)90009-9 3525819

[121] MRC Vitamin Study Research Group Prevention of neural tube defects: Results of the Medical Research Council Vitamin Study. Lancet. 1991;338:131–7. 10.1016/0140-6736(91)90133-A 1677062

[122] Czeizel AE Dudas I Prevention of the first occurrence of neural-tube defects by periconceptional vitamin supplementation. N Engl J Med. 1992;327:1832–5. 10.1056/NEJM199212243272602 1307234

[123] Crellin R Bottiglieri IT Reynolds EH Folates and Psychiatric Disorders Clinical Potential. Drugs. 1993;45:623–36. 10.2165/00003495-199345050-00001 7686459

[124] Bleyer WA Drake JC Chabner BA Neurotoxicity and elevated cerebrospinal fluid methotrexate concentration in meningeal leukaemia. N Engl J Med. 1973;289:770–3. 10.1056/NEJM197310112891503 4517004

[125] Kay HE Knapton PJ O’Sullivan JP Wells DG Harris RF Innes EM Encephalopathy in acute leukaemia associated with methotrexate therapy. Arch Dis Child. 1972;47:344–54. 10.1136/adc.47.253.344 4504035 PMC1648136

[126] Botez M, Reynolds E. Folic acid in neurology, psychiatry and internal medicine. New York, USA: Raven Press; 1979.

[127] Shorvon SD Carney MW Chanarin I Reynolds EH The neuropsychiatry of megaloblastic anaemia. BMJ. 1980;281:1036–8. 10.1136/bmj.281.6247.1036 6253016 PMC1714413

[128] Patanwala I King MJ Barrett DA Rose J Jackson R Hudson M Folic acid handling by the human gut: Implications for food fortification and supplementation. Am J Clin Nutr. 2014;100:593–9. 10.3945/ajcn.113.080507 24944062 PMC4095662

[129] Spector R Levy P Abelson H Identification of dihydrofolate reductase in rabbit brain. Biochem Pharmacol. 1977;26:1507–11. 10.1016/0006-2952(77)90423-3 901568

[130] Krsička D Geryk J Vlčková M Havlovicová M Macek M Pourová R Identification of likely associations between cerebral folate deficiency and complex genetic- and metabolic pathogenesis of autism spectrum disorders by utilization of a pilot interaction modeling approach. Autism Res. 2017;10:1424–35. 10.1002/aur.1780 28339176

[131] Frye RE Slattery JC Quadros EV Folate metabolism abnormalities in autism: Potential biomarkers. Biomark Med. 2017;11:687–99. 10.2217/bmm-2017-0109 28770615

[132] Ramaekers VT Hausler M Opladen T Heimann G Blau N Psychomotor retardation, spastic paraplegia, cerebellar ataxia and dyskinesia associated with low S-methyltetrahydrofolate in cerebrospinal fluid: A novel neurometabolic conditions responding to folinic acid substitution. Neuropediatrics. 2002;33:301–8. 10.1055/s-2002-37082 12571785

[133] Ramaekers V Thony B Sequeira J Ansseau M Philippe P Boemer F Folinic acid treatment for schizophrenia associated with folate receptor autoantibodies. Mol Genet Metab. 2014;113:307–14. 10.1016/j.ymgme.2014.10.002 25456743

[134] Sadighi Z Butler I Koenig M Adult-onset cerebral folate deficiency. Arch Neurol. 2012;69:778–9. 10.1001/archneurol.2011.3036 22371854

[135] Vasconcelos C Perry IS Gottfried C Riesgo R Castro K Folic acid and autism: updated evidences. Nutr Neurosci. 2025;28:273–307. 10.1080/1028415X.2024.2367855 38968136

[136] Roufael M Bitar T Sacre Y Andres C Hleihel W Folate–methionine cycle disruptions in ASD patients and possible interventions: A systematic review. Genes (Basel). 2023;14:709. 10.3390/genes14030709 36980981 PMC10048251

[137] Ramaekers VT Rothenberg SP Sequeira JM Opladen T Blau N Quadros EV Autoantibodies to folate receptors in the Cerebral Folate Deficiency Syndrome. N Engl J Med. 2005;352:1985–91. 10.1056/NEJMoa043160 15888699

[138] Rossignol DA Frye RE Cerebral folate deficiency, folate receptor alpha autoantibodies and leucovorin (Folinic acid) treatment in autism spectrum disorders: A systematic review and meta-analysis. J Pers Med. 2021;11:1141. 10.3390/jpm11111141 34834493 PMC8622150

[139] Luo T Li K Ling Z Zhao G Li B Wang Z De novo mutations in folate-related genes associated with common developmental disorders. Comput Struct Biotechnol J. 2021;19:1414–22. 10.1016/j.csbj.2021.02.011 33777337 PMC7966843

[140] Rice D Barone S Critical periods of vulnerability for the developing nervous system: Evidence from humans and animal models. Environ Health Perspect. 2000;108 Suppl3:511–33. 10852851 10.1289/ehp.00108s3511 PMC1637807

[141] Zeisel SH The supply of choline is important for fetal progenitor cells. Semin Cell Dev Biol. 2011;22:624–8. 10.1016/j.semcdb.2011.06.002 21693194 PMC3188336

[142] LaSalle JM A genomic point-of-view on environmental factors influencing the human brain methylome. Epigenetics. 2011;6:862–9. 10.4161/epi.6.7.16353 21617367 PMC3154427

[143] Kong L Chen X Gissler M Lavebratt C Relationship of prenatal maternal obesity and diabetes to offspring neurodevelopmental and psychiatric disorders: a narrative review. Int J Obes (Lond). 2020;44:1981–2000. 10.1038/s41366-020-0609-4 32494038 PMC7508672

[144] Merikangas AK Almasy L Using the tools of genetic epidemiology to understand sex differences in neuropsychiatric disorders. Genes Brain Behav. 2020;19:e12660. 10.1111/gbb.12660 32348611 PMC7507200

[145] Williams OOF Coppolino M George SR Perreault ML Sex differences in dopamine receptors and relevance to neuropsychiatric disorders. Brain Sci. 2021;11:1199. 10.3390/brainsci11091199 34573220 PMC8469878

[146] Seedat S Scott KM Angermeyer MC Berglund P Bromet EJ Brugha TS Cross-national associations between gender and mental disorders in the World Health Organization world mental health surveys. Arch Gen Psychiatry. 2009;66:785. 10.1001/archgenpsychiatry.2009.36 19581570 PMC2810067

[147] Werling DM Geschwind DH Understanding sex bias in autism spectrum disorder. Proc Natl Acad Sci U S A. 2013;110:4868. 10.1073/pnas.1301602110 23476067 PMC3612630

[148] Iqbal J Di Huang G Xue YX Yang M Jia XJ Role of estrogen in sex differences in memory, emotion and neuropsychiatric disorders. Mol Biol Rep. 2024;51:415. 10.1007/s11033-024-09374-z 38472517

[149] Eikelboom WS Pan M Ossenkoppele R Coesmans M Gatchel JR Ismail Z Sex differences in neuropsychiatric symptoms in Alzheimer’s disease dementia: a meta-analysis. Alzheimers Res Ther. 2022;14:48. 10.1186/s13195-022-00991-z 35379344 PMC8978393

