# Supplementary material for: Folate and global health: part 5. syntheses on neuropsychiatric disorders
Source: J Glob Health. 2026 Aug 17;16:04286. doi: 10.7189/jogh.16.04286 (PMC13480459; doi:10.7189/jogh.16.04286)
Supplement: Supplementary file 1 [file jogh-16-04286-s001.pdf]

Supplement to: Yoo S, Montazeri A, Bennett D, Bo Y, Chen P, Duthie S, Jensen N, Kaminga A, Lai J, Li X, MacFarlane A, Martinez H, McNulty H, Momoli F, Mossey P, Munger R, Parajuli RP, Kent MP, Rubini M, Senekal M, Sikora L, Stintzi A, Theodoratou E, Wang H, Yaktine A, Little J. Folate and global health review series, part 4: syntheses on folate and autoimmune diseases and skeletal outcomes. J Glob Health. 2026;16:04286.

Table S1. Characteristics of all included evidence syntheses examining the relationship between folate intake/status and neuropsychiatric disorders (chronological)

First author (year)	Synthesis type	Study population	Folate exposure	Outcome	No. of studies (Design)	Total (Case)	Country	Comparators
**Neurological conditions**								
Ellinson et al. (2004)	SR	Individuals with AD or VD	Serum folate	Cognitive function	1 (PC)	322 (195)	NR	Case vs control
		Older adults		AD	2 (CC)	370 (NR)		High vs low
Schneider et al. (2006)	SR	Older adults	Total folate	Cognitive function	16 (CS)	NR (NR)	NR	Case vs control
			Total folate	AD	4 (CC)	NR (NR)		
			Total folate	Dementia/MCI	3 (CC)	NR (NR)		
			Serum/RBC folate	Cognitive function	5 (PC)	1,020 (NR)	NR	High vs low
			Serum/RBC folate	AD/dementia	4 (PC)	3,094 (NR)		
			Dietary intake	Cognitive function	3 (PC)	4,638 (NR)	NR	High vs low
			Dietary intake	AD	1 (PC)	579 (NR)		
			FA supplement	Cognitive function	2 (RCT)	220 (NR)	NR	Placebo
Balk et al. (2007)	SR	Older adults with or without cognitive impairment or dementia	FA supplement (0.75-20.0 mg/d for 35-70 days)	Cognitive function	3 (RCT)	39 (NR)	NR	Placebo
Raman et al. (2007)	SR	Older adults	Plasma/serum folate	Cognitive function	8 (PC), 1 (RC), 1 (CC)	2,910 (NR)	US, Canada, Norway, Netherlands, Sweden, Switzerland	High vs low
		Older adults	Plasma/serum folate	AD	5 (PC), 4 (CC)	3,886 (859)	Canada, Switzerland, Sweden, Italy, US, Israel, UK	High vs low
Jia et al. (2008)	SR	Individuals with mild/moderate dementia or cognitive decline and healthy controls	FA supplement (750 µg.d-20 mg/d for 35 days-10 weeks, or 5-MTHF 50 mg/d for 8 weeks)	Cognitive function	4 (RCT)	NR (NR)	Italy, Australia, US	Placebo
Purnell et al. (2009)	SR	General population	Serum folate	AD	2 (PC)	1,908 (NR)	Italy, US	High vs low
			Dietary intake		1 (PC)	965 (NR)	US	High vs low
Van Dam et al. (2009)	MA	Individuals with AD and healthy controls	Serum folate	AD	6 (CC)	699 (NR)	NR	Case vs control

Vogel et al. (2009)	SR	Individuals with cognitive impairment or dementia	FA supplement	Cognitive function (memory scores, information processing speed, sensory-motor speed)	4 (RCT), 1 (PC)	NR (NR)	NR	Placebo
Ranganathan et al. (2009)	SR	Individuals with epilepsy	FA supplement (5-15 mg/d for 3-6.5 months)	Intelligence	4 (RCT)	219 (NR)	UK, US	Placebo/none
			FA supplement (5-15 mg/d for 3-6 months)	Behavior	3 (RCT)	149 (NR)	UK	
	MA	Individuals with epilepsy	FA supplement (15 mg/d for 3-6.5 months)	Seizure frequency	2 (RCT)	75 (NR)	UK	Placebo/none
			FA supplement (4 mg/d for 6 months)	Gingival index	2 (RCT)	28 (NR)	US	
			FA supplement (4 mg/d for 6 months)	Plaque index	2 (RCT)	28 (NR)	US	
			FA supplement (4 mg/d for 6 months)	Hyperplasia index	2 (RCT)	28 (NR)	US	
Wald et al. (2010)	MA	Older adults without dementia at baseline	FA supplement (0.75-15 mg/d for 1-36 months)	Cognitive function	4 (RCT)	1,083 (NR)	Italy, UK, Netherlands, Australia	Placebo
Lohner et al. (2012)	SR	Infants with Down syndrome	FA supplement (0.1 mg/d-1 mg/kg/d)	Cognitive function in infants with Down syndrome	2 (RCT)	159 (NR)	NR	Placebo
Perez et al. (2012)	SR	Older adults	Serum folate	Vascular dementia<br>AD	1 (PC), 1 (CS)<br>1 (PC), 1 (CS)	2,703 (142)<br>2,824 (263)	NR	Case vs control
Michelakos et al. (2013)	MA	Older adults	Serum folate	Cognitive function (MMSE-Greek)	3 (PC), 10 (CS)	9,747 (NR)	Spain, UK, Italy, Switzerland, Netherlands, Greece, US, Canada	Case vs control
Shah et al. (2013)	SR	Older adults	Dietary intake/total folate	AD	3 (PC)	7,068 (NR)	NR	High vs low
			Plasma/serum folate	AD/dementia	1 (PC), 1 (CC)	433 (NR)		
			Plasma/serum folate	Cognitive function	1 (PC), 2 (CS)	4,066 (NR)		
Barnes et al. (2014)	SR	Older adults	Plasma/serum folate	Cognitive function	2 (PC)	1,058 (NR)	US, Netherlands, Italy (not fully reported)	High vs low
			FA supplement	Memory, information processing speed, attention efficiency	3 (RCT)	853 (NR)		Placebo
Lopes et al. (2014)	MA	Individuals with AD and healthy controls	Plasma folate	AD	31 (CS, CC)	4,555 (2,108)	NR	Case vs control

Cooper et al. (2015)	SR	Individuals with MCI	Serum folate	Progression to dementia of any type	3 (PC)	301 (NR)	Austria, Italy, Poland	High vs low
Shen et al. (2015)	MA	Individuals with PD and healthy controls	Plasma/serum folate	PD	10 (CC)	1,659 (NR)	NR	Case vs control
			Dietary intake		3 (CC)	NR (NR)		
Shen et al. (2015)	MA	Individuals with AD and healthy controls	Plasma folate	AD	32 (CC)	5,143 (NR)	NR	Case vs control
			CSF folate		4 (CC)	295 (NR)		
Colapinto et al. (2016)	SR	General population	Serum/RBC folate	Cognitive impairment	1 (RCT)<br>7 (PC)	369 (NR)<br>12,254 (NR)	NR	High vs low
De Wilde et al. (2017)	MA	Individuals with AD and healthy controls	CSF folate	AD	9 (CC)	845 (307)	NR	Case vs control
Xie et al. (2017)	MA	Individuals with or without PD	Plasma folate	PD	9 (CS)	981 (NR)	NR	High vs low
Murray et al. (2019)	SR	Individuals with or without PD	Dietary intake	PD	1 (PC), 1 (CC)	136,674 (NR)	US, Japan	High vs low
Vlachos et al. (2019)	SR	Community-dwelling older adults with diagnosed MCI or AD	FA supplement (1-1.25 mg/d or 400 μg/d for 6 months)	Cognitive function (MMSE, IQ/WAIS-RC, ADL/IADL)	3 (RCT)	399 (NR)	UK, China	Placebo
Dong et al. (2020)	MA	Chinese individuals with PD and healthy controls	Plasma folate	PD	25 (CC)	2,808 (1,705)	China	Case vs control
Widyadharma et al. (2020)	SR	Individuals with MCI or AD with MMSE scores 3-26	FA supplement (1.25 mg/d for 6 months, 400 μg/d for 12 months)	Cognitive function (MMSE, IQ, digit span)	2 (RCT)	273 (NR)	China	Placebo
Liampas et al. (2020)	MA	Individuals with migraine and healthy controls	Serum folate	Migraine	11 (CC)	1,406 (744)	Italy, Turkey, Austria, Korea, Japan, China	Case vs control
			Serum folate	Migraine with aura	4 (CC)	405 (144)	Turkey, Korea, Japan	Case vs control
		Individuals with migraine	Serum folate	Migraine with aura vs without aura	4 (CC)	292 (144)	Turkey, Korea, Japan	With aura vs without aura
Asadi-Pooya et al. (2021)	SR	Individuals with epilepsy of all types	FA supplement<br>Adults: 5-20 mg/d for 3-6 months<br>Children: 5-10 mg/d for 3 months	Seizure frequency	7 (intervention trials)	167 (NR)	Denmark, UK, Canada, US, India	Placebo
Wang Q et al. (2021)	MA	Older adults	Plasma/serum folate	AD	37 (PC, CC)	7,833 (NR)	NR	High vs low
				Vascular dementia	11 (PC, CC)	5,209 (NR)		
Zhang et al. (2021)	MA	Individuals with or without AD	Plasma/serum folate	AD	52 (CS)	7,814 (NR)	NR	High vs low
			Plasma/serum folate		15 (CC)	2,030 (NR)		
			Plasma/serum folate		5 (PC)	1,835 (NR)		
			Total intake		11 (PC)	4,325 (NR)		

Gil Martinez et al. (2022)	SR	Older adults with MCI or AD	FA supplement (1.25 mg/d for 6 months, 400 µg/d for 6-24 months)	Cognitive function (FSIQ, WAIS-RC, MMSE)	4 (RCT)	649 (NR)	China	Placebo or conventional treatment
Huang et al. (2022)	SR	Chinese older adults with MCI or AD	FA supplement (1.25 mg/d for 6 months or 0.4 mg/d for 24 months)	Cognitive function	2 (RCT)	341 (NR)	China	Placebo
Wang Z et al. (2022)	MA	Older adults with or without dementia	Dietary intake	Incident dementia	5 (PC)	13,473 (NR)	France, US	High vs low
			Plasma/serum folate	Dementia	3 (PC), 6 (CS)	6,654 (NR)	UK, Finland, US, China, Italy, Korea	
Hu et al. (2023)	MA	Individuals with ALS and healthy controls (matched on age and sex)	Plasma folate	ALS	3 (CC)	338 (169)	Italy, China	Case vs control
**Psychiatric disorders**								
Frazer et al. (2005)	SR	Older individuals with depression	Methylfolate	Depressive symptoms	2 (RCT) 1 (pre-post trial)	NR (NR)	NR	Placebo
Harbottle et al. (2008)	SR	Individuals with depression	FA supplement	Depression	3 (RCT)	247 (NR)	NR	Placebo
			Plasma folate		3 (CC)	NR (NR)	NR	High vs low
Morris et al. (2008)	SR	Individuals with MDD and healthy controls	Plasma/serum folate	MDD symptoms	12 (CC, CS, PC)	NR (NR)	NR	Case vs control
		Individuals with MDD	Plasma/serum folate	MDD treatment response	5 (open-label trials)	548 (NR)	NR	Placebo
			FA supplement	MDD treatment response	2 (RCT), 2 (open-label trials)	NR (NR)	NR	Placebo
Main et al. (2010)	SR	Children with ASD and healthy controls	Serum folate	ASD	5 (CC)	416 (89)	US, Germany	Case vs control
Murakami et al. (2010)	SR	General population	Dietary intake	Depressive symptoms	3 (PC), 5 (CS)	22,522 (NR)	Netherlands, Finland, Spain, France, US, Japan	High vs low
Taylor et al. (2010)	MA	Individuals with MDD	FA supplement (500 µg/d, 15 mg/d methylfolate)	Depressive symptoms (HDRS)	2 (RCT)	124 (NR)	UK	Placebo or alternative treatment
Nahas et al. (2011)	SR	Individuals with depression	FA supplement (0.2 mg/d for 1 year or 0.5 mg/d for 10 weeks, or l-methylfolate 15 mg/d for 6 months)	Depressive symptoms	3 (RCT)	204 (NR)	NR	Placebo

Sarris et al. (2011)	SR	Individuals taking lithium or individuals with bipolar-I disorder with current acute mania	FA supplement (200 IU, 3 mg)	Bipolar depression symptom (BDI-AMI or YMRS)	2 (RCT)	105 (NR)	NR	Placebo
Dhingra et al. (2012)	SR	Individuals with depression	FA supplement (0.2 mg/d for 1 year or 0.5 mg/d for 10 weeks)<br>L-methylfolate (15 mg/d for 6 months)	Depressive symptoms (Beck Depression Scale, Hamilton Rating Scale for Depression)	3 (RCT)	204 (NR)	NR	Placebo
Frustaci et al. (2012)	MA	Children with ASD and healthy controls	Plasma/serum folate<br>RBC folate	ASD	5 (CS)<br>1 (CS)	314 (NR)<br>33 (NR)	NR	Case vs control
Sanhueza et al. (2013)	SR	General population without current or history of depression at baseline	Dietary intake	Unipolar depression	2 (PC)	4,177 (NR)	Finland, France	High vs low
Chia et al. (2015)	SR	Individuals with schizophrenia	Methylfolate supplement (15 mg/d) or FA (2 mg/d) for 8 weeks-6 months	Schizophrenia symptoms	2 (RCT)	49 (NR)	US, UK	Placebo
Cao et al. (2016)	MA	Individuals with schizophrenia and healthy controls	Serum folate	Schizophrenia	20 (CC)	2,739 (1,463)	Netherland, Spain, Poland, Greece, Serbia, China, Korea, Iran, Turkey, Tunisia, Mexico	Case vs control
Castro et al. (2016)	SR	Mother-offspring pairs	FA supplement (folinic acid 1 mg/kg/d then 2-3 mg/kg/d)	ASD	1 (RCT)	25 (NR)	NR	Placebo/none
			Maternal FA		4 (CC, PC)	101,187 (NR)	Oman, US	Placebo/none
			Dietary folate		4 (CC, PC)	468 (NR)	Oman, US, China	High vs low
			Serum folate		1 (CC)	80 (NR)	Oman	Case vs control
Sarris et al. (2016)	MA	Individuals with diagnosed depression taking antidepressants	FA supplement (0.5-10 mg/d for 6-12 weeks)	Depressive symptoms (HAM-D 17)	3 (RCT), 1 (open-label trial)	671 (NR)	NR	Placebo
	SR		Methylfolate or folinic acid (15-30 mg/d for 1-6 months)		2 (RCT), 1 (open-label trial)	269 (NR)	NR	Placebo

Bender et al. (2017)	MA	Individuals with depression or depressive symptoms and healthy controls	Dietary folate (11) Serum folate (30) RBC folate (7)	Depression	48 (CC)	35,801 (8,519)	Netherlands, UK, Germany, Ireland, US, Canada, Australia, Finland, Japan, Korea, China, Singapore, Israel, Turkey, Greece, UAE, India, Nigeria, South Africa	Case vs control
Firth et al. (2017)	SR	Individuals with schizophrenia	FA supplement (2.0 mg/d) or methylfolate supplement (15 mg/d) for 12-24 weeks	Schizophrenia symptoms	2 (RCT)	45 (NR)	UK, US	Placebo
Schefft et al. (2017)	MA	Individuals with depression taking antidepressants	FA supplement (0.5-10 mg/d for 6-12 weeks)	Unipolar depression symptoms (HDRS or MADRS)	3 (RCT)	567 (NR)		Placebo
Sparling et al. (2017)	SR	Pregnant women	Dietary intake	Perinatal depression	3 (PC)	10,530 (NR)	UK, Japan	High vs low
					1 (CS)	1,277 (NR)	Korea	
Wang et al. (2017)	MA	Mother-offspring pairs	FA supplement before and during pregnancy	ASD	5 (PC)	616,911 (NR)	Norway, Netherlands, US, China	High vs low
					11 (CC)	14,994 (NR)	US, China, Oman	Case vs control
Firth et al. (2018)	MA	Individuals with first episode psychosis and healthy controls	Plasma/serum/RBC folate	First-episode psychosis symptoms	6 (CS)	827 (421)	NR	Case vs control
Sakuma et al. (2018)	MA	Individuals with schizophrenia	FA supplement (2-500 mg/d for 12-16 weeks)	Total symptoms	4 (RCT)	121 (NR)	UK, Finland, Italy, US	Placebo
			FA supplement (2-500 mg/d for 12-26 weeks)	Negative symptoms	3 (RCT)	104 (NR)	UK, Finland, Italy, US	Placebo
Trincado et al. (2018)	MA	Individuals with MDD	FA supplement	Depressive symptoms	4 (RCT)	591 (NR)	NR	Placebo
Trujillo et al. (2018)	SR	Pregnant women	Plasma/serum folate	Antenatal depression	1 (PC), 1 (CS)	1,053 (NR)	Japan, Singapore	High vs low
			Plasma/RBC folate	Postpartum depression	2 (PC)	3,823 (NR)	UK, Singapore	High vs low
Guo et al. (2019)	MA	Mother-offspring pairs	Maternal FA supplement	ASD	2 (PC)	144,865 (2,198)	Norway, Sweden	Placebo/none

Hsieh et al. (2019)	MA	Individuals with bipolar disorder and healthy controls	Serum folate	Bipolar disorder	6 (CC)	1,241 (481)	Spain, Israel, Turkey, Tunisia, Malaysia	Case vs control
Iglesias Vazquz et al. (2019)	MA	Mother-offspring pairs	Maternal FA	Mental development	2 (RCT) 4 (PC)	11,302 (NR)	Spain, Poland, Greece, Hungary	Placebo/none
				Motor development	4 (PC)	8,805 (NR)	Spain, Poland, Greece	Placebo/none
				ASD	6 (PC), 3 (CC)	739,226 (6,396)	Norway, Denmark, Sweden, Israel, US	Placebo/none
Zhong et al. (2020)	SR	Mother-offspring pairs	Maternal FA supplement	ASD	9 (PC)	383,811 (NR)	Norway, Netherlands, Denmark, Sweden, US, Israel	High vs low
					3 (CC)	2,052 (1,219)	US, China	Case vs control
			Maternal plasma folate	ASD	3 (PC)	106,412 (NR)	US, Norway	High vs low
					1 (CC)	200 (100)	Sweden	Case vs control
Balandeh et al. (2021)	MA	Individuals with obsessive compulsive disorder and healthy controls	Serum folate	Obsessive compulsive disorder	4 (CC)	245 (140)	Turkey, Israel	Case vs control
Gallardo-Carrasco et al. (2021)	MA	Children with ASD	Serum folate	ASD	4 (CC)	299 (148)	Turkey, China	Case vs control
Hoxha et al. (2021)	SR	Mother-offspring pairs	Maternal FA	ASD	2 (PC)	603,283 (NR)	NR	Placebo/none
		Children with ASD and neurotypical controls	Serum/CSF folate		2 (RCT), 1 (CR), 1 (CC)	3,064 (NR)	NR	High vs low
Sampaio et al. (2021)	SR	Mother-offspring pairs	Maternal plasma folate	ASD	17 (NR)	NR (NR)	Netherlands, Denmark, Norway, US, Colombia, China, Palestine	Placebo/none
			Maternal FA supplement					
Vander Burg et al. (2021)	SR	Outpatients with MDD	FA supplement (5 mg/d for 12 weeks, or folinic acid 15 mg/d for 2 weeks then 30 mg/d for 6 weeks)	MDD	2 (RCT)	497 (NR)	UK, US	Placebo
		Outpatients with schizophrenia	FA supplement (2 mg/d for 12 weeks, or methylfolate 15 mg/d for 12-24 weeks)	Schizophrenia	2 (RCT)	87 (NR)	US	Placebo

Jin et al. (2022)	MA	Pregnant women	Serum/RBC folate	Perinatal depression (antenatal or postpartum)	6,442 (NR)	5 (PC)	UK, US, Singapore, India	High vs low
			FA supplement		12,881 (NR)	4 (PC), 3 (CS)	UK, China	Placebo/none
Liu et al. (2022)	MA	Mother-offspring pairs	Maternal FA supplement	ASD	693,757 (5,755)	3 (PC), 1 (CC)	Sweden, Italy, Norway	High vs low
Prades et al. (2022)	MA	Individuals with ASD and healthy controls	Plasma/serum folate	ASD	12 (CS)	1,665 (710)	Norway, US, Romania, Oman, Turkey, China	Case vs control
		Individuals with ADHD and healthy controls		ADHD	5 (CS)	1,121 (537)	Turkey, India, Taiwan	Case vs control
Yan et al. (2022)	MA	Individuals with OCD and healthy controls	Plasma/serum folate	OCD	5 (CC)	309 (NR)	Turkey, Iran, Israel	Case vs control
Borges-Vieira et al. (2023)	SR	Individuals with depression	FA supplement (5 mg/d for 12 weeks) Methylfolate supplement (15 mg/d for 4 or 24 weeks)	Depression (HAM-D, MADRS, QIDS-SR)	3 (RCT)	923 (NR)	NR	Placebo
Gabriel et al. (2023)	SR	Individuals with bipolar disorder	FA supplement (3 mg/d for 3 weeks or 15 mg/d methylfolate for 6 weeks)	Bipolar disorder symptoms	1 (RCT), 1 (open-label trial)	98 (NR)	NR	Placebo

AD: Alzheimer's disease; ADHD: attention deficit and hyperactivity disorder; ALS: amyotrophic lateral sclerosis; AS: Asperger's syndrome; ASD: autism spectrum disorder; CC: case-control study; CR: case report; CS: cross-sectional study; CSF: cerebrospinal fluid ; FA: folic acid; MA: meta-analysis; MCI: mild cognitive impairment; PC: prospective cohort; PD: Parkinson's disease; PDD-NOS: pervasive developmental disorder – not otherwise specified; RBC: red blood cell; RC: retrospective cohort; RCT: randomized controlled trial; SR: systematic review; VD: vascular dementia

Table S2. Summary of the meta-analyses reporting the association of folate intake/status and risk of neuropsychiatric disorders

Author (year)	Outcome	Study population/ subgroup	No. studies (design)	Dose, duration, follow-up (mean, range)	No. total (case)	Comparator	Summary effect ()	I^2	P_{Egger}
**Epilepsy – FA supplement**									
Ranganathan (2009)	Seizure frequency	Individuals with epilepsy	2 (RCT)	15 mg/d for 3-6.5 months	75 (18)	Placebo	OR=0.98 (0.32, 2.98)	0% (p=0.71)	NR
**ALS – Plasma folate**									

Hu (2023)	ALS	Individuals with ALS	3 (CC)	NA	338 (169)	Healthy control	MD= -0.52 ng/mL (-1.89, 0.84)	83%	NR
**Migraine - Serum folate**									
Liampas (2020)	Migraine	General population	11 (CC)	NA	1,406 (744)	Healthy control	**SMD= -0.36 (-0.68, -0.05)**	87% (p<0.1)	Yes
		- Adults only	8 (CC)	NA	1,215 (643)	Healthy control	SMD= -0.11 (-0.25, 0.01)	6% (p=0.39)	Yes
	Migraine with aura	Individuals with migraine	4 (CC)	NA	405 (144)	Healthy control	SMD= -0.17 (-0.44, 0.10)	47% (p=0.13)	NR
			4 (CC)	NA	292 (144)	Migraine without aura	SMD= -0.16 (-0.35, 0.04)	44% (p=0.15)	NR
**Alzheimer's disease – Plasma/serum folate**									
Van Dam (2009)	AD	General population	6 (CC)	NA	699 (312)	Healthy control	**SMD= 0.56 (0.34, 0.95)**	NR	NR
Lopes (2014)	AD	General population	31 (CS, CC)	NA	4,555 (2,108)	Healthy control	**MD= -4.33 (-5.83, -2.84)**	99% (p<.001)	NR
Shen (2015)	AD	General population	32 (CC)	NA	5,143 (2,446)	Healthy control	**SMD= -0.83 nmol/L (-1.08, -0.57)**	93.9% (p=0.000)	NR
Wang Q (2021)	AD	Older individuals	37 (PC, CC)	NA	7,833 (2,932)	Healthy control	SMD= -0.50 (-0.64, -0.36)	85.5% (p=0.000)	NR
Zhang X (2021)	AD	General population	52 (CS)	NA	7,814 (3,496)	Healthy control	**SMD= -0.60 (-0.65, -0.55)**	97.2% (p=0.000)	No
Zhang X (2021)	AD	General population	15 (CC)	NA	2,030 (971)	Healthy control	OR=0.96 (0.93, 0.99)	46.6% (p=0.02)	No
		- Folate non-deficient (≥ 13.5 nmol/L)	3 (CC)	NA	401 (238)	Healthy control	OR=0.86 (0.46, 1.26)	0% (p=0.56)	No
		- Folate deficient (< 13.5 nmol/L)	6 (CC)	NA	885 (379)	Healthy control	OR=1.94 (1.02, 2.86)	0% (p=0.82)	No
Zhang X (2021)	AD	Older adults with folate deficiency or possible deficiency	5 (PC)	NA	1,835 (NR)	Deficient vs normal	**RR=1.88 (1.20, 2.57)**	0% (p=0.98)	No
**Alzheimer's disease – CSF folate**									
Shen (2015)	AD	General population	4 (CC)	NA	295 (150)	Healthy control	**SMD= -0.86 nmol/L (-1.29, -0.43)**	58.7% (p=0.06)	NR
De Wilde (2017)	AD	General population	9 (CC)	NA	845 (307)	Healthy control	**MD= -0.56 (-0.73, -0.38)**	NR	NR
**Alzheimer's disease – Plasma/CSF folate**									
Shen (2015)	AD	General population	36 (CC)	NA	5,438 (2,596)	Healthy control	**SMD= -0.84 nmol/L (-1.07, -0.60)**	93.9% (p=0.000)	NR
		- <74 years	14 (CC)	NA	1,650 (806)	Healthy control	**SMD= -0.63 nmol/L (-0.96, -0.30)**	89.3% (p=0.000)	NR
		- ≥75 years	16 (CC)	NA	2,942 (1,326)	Healthy control	**SMD= -0.99 nmol/L (-1.38, -0.59)**	95.1% (p=0.000)	NR
**Alzheimer's disease – Total folate intake**									
	AD	Older adults	11 (PC)	NA	4,325 (NR)	High vs low	**RR=0.50 (0.25, 0.76)**	32.9% (p=0.20)	No

Zhang X (2021)		- < 400 µg/d	2 (PC)	NA	426 (NR)	High vs low	RR=1.15 (0.28, 2.02)	0% (p=0.45)	No
		- ≥ 400 µg/d	3 (PC)	NA	608 (NR)	High vs low	**RR=0.44 (0.18, 0.71)**	35.3% (p=0.21)	No
**Parkinson's disease – Dietary folate**									
Shen (2015)	PD	General population	3 (CC)	NA	NR (NR)	Healthy control	OR=1.01 (0.68, 1.34)	0% (p=0.46)	NR
**Parkinson's disease – Plasma/serum folate**									
Shen (2015)	PD	General population	10 (CC)	NA	1,659 (735)	Healthy control	SMD= -0.12 nmol/L (-0.28, 0.04)	49.2% (p=0.04)	NR
		- Levodopa treated group	8 (CC)	NA	1,406 (692)	Healthy control	SMD= -0.14 nmol/L (-0.33, 0.04)	58.5% (p=0.02)	NR
		- Levodopa untreated group	2 (CC)	NA	253 (43)	Healthy control	SMD= 0.02 nmol/L (-0.31, 0.35)	0% (p=0.84)	NR
Xie (2017)	PD	General population	9 (CS)	NA	981 (379)	Healthy control	**SMD= -0.21 (-0.34, -0.08)**	0% (p=0.80)	No
Dong (2020)	PD	Chinese individuals with PD and healthy controls	25 (CC)	NA	2,808 (1,705)	Healthy control	**SMD= -0.31 (-0.47, -0.15)**	74.3% (p=0.00)	NR
**Dementia – Dietary intake**									
Wang Z (2022)	Incident dementia	Older adults	5 (PC)	NA	13,473 (NR)	High vs low	**HR=0.61 (0.47, 0.78)**	39% (p=0.16)	No
**Dementia – Plasma/serum folate**									
Wang Q (2021)	Vascular dementia	Older adults	11 (PC, CC)	NA	5,209 (308)	Healthy control	**SMD= -0.80 (-1.2, -0.39)**	82.6 (p=0.000)	NR
Wang Z (2022)	Dementia	Older adults	3 (PC), 6 (CS)	NA	6,654 (NR)	Low vs high	**OR=1.76 (1.24, 2.50)**	81% (p<.00001)	No
**Alzheimer's disease or Vascular dementia – Plasma/serum folate**									
Wang Q (2021)	AD or VaD	Older adults	37 (CC), 5 (PC)	NA	8,141 (3,240)	Healthy control	**SMD= -0.57 nmol/L (-0.71, -0.43)**	85.8% (p=0.000)	p=0.00
		- Caucasians	38 (PC, CC)	NA	5,553 (2,403)	Healthy control	**SMD= -0.68 nmol/L (-0.86, -0.50)**	87.8% (p=0.000)	NR
		- Asians	10 (PC, CC)	NA	2,992 (837)	Healthy control	**SMD= -0.26 nmol/L (-0.38, -0.31)**	46.2% (p=0.000)	NR
		- 60-70 years	3 (PC, CC)	NA	1,280 (312)	Healthy control	**SMD= -0.26 nmol/L (-0.49, -0.04)**	85.8% (p=0.000)	NR
		- 70-80 years	38 (PC, CC)	NA	6,427 (2,639)	Healthy control	**SMD= -0.57 nmol/L (-0.73, -0.42)**	85.4% (p=0.000)	NR
		- 80-90 years	7 (PC, CC)	NA	938 (340)	Healthy control	**SMD= -0.69 nmol/L (-1.19, -0.19)**	90.5% (p=0.000)	NR
		- Male	12 (PC, CC)	NA	1,100 (505)	Healthy control	**SMD= -0.64 nmol/L (-0.97, -0.3)**	84.1% (p=0.000)	NR
		- Female	27 (PC, CC)	NA	6,077 (2,021)	Healthy control	**SMD= -0.57 nmol/L (-0.74, -0.39)**	85.4% (p=0.000)	NR
		- Prospective cohorts only	5 (PC)	NA	NR (NR)	Low vs high	**RR=1.73 (1.22, 2.34)**	0% (p=0.77)	NR
**Cognitive function – FA supplement**									

Wald (2010)	Cognitive function	Older adults without dementia at baseline	4 (RCT)	0.75-15 mg/d for 1-36 months	1,083 (NA)	Placebo	SMD= 0.10 (-0.06, 0.25)	NR	NR
**Cognitive function – Serum folate**									
Michelakos (2013)	Cognitive impairment	Older adults	3 (PC), 10 (CS)	NA	9,747 (NR)	Low vs high	**OR=1.66 (1.40, 1.96)**	NR	NR
		- Male	2 (PC), 6 (CS)	NA	2,300 (NR)	Low vs high	**OR=1.95 (1.55, 2.46)**	NR	NR
		- Female	2 (PC), 6 (CS)	NA	3,115 (NR)	Low vs high	**OR=1.90 (1.55, 2.32)**	NR	NR
		- PC only	3 (PC)	NA	3,103 (NR)	Low vs high	**OR=1.40 (1.06, 1.84)**	NR	NR
		- CS only	10 (CS)	NA	6,644 (NR)	Low vs high	**OR=1.69 (1.40, 2.04)**	NR	NR
**Depression – FA supplement**									
Taylor (2010)	Depressive symptoms (HDRS)	Individuals with MDD	2 (RCT)	FA 500 µg/d or methylfolate 15 mg/d	124 (NA)	Placebo or antidepressant alone	**MD= -2.65 (-4.93, -0.38)**	0% (p=0.91)	NR
Sarris (2016)	Depressive symptoms (HAM-D 17)	Individuals diagnosed with depression and taking antidepressants	3 (RCT), 1 (open-label trial)	FA 0.5-10 mg/d for 6-12 weeks	671 (NA)	Antidepressant alone	SMD= 0.49 (-0.31, 1.29)	93% (p<.001)	No
Schefft (2017)	Depressive symptoms (HDRS or MADRS)	Individuals with depression taking antidepressants	3 (RCT)	FA 0.5-10 mg/d for 6-12 weeks	567 (NA)	Antidepressant alone	SMD= -0.26 (-0.67, 0.15)	68% (p=0.05)	NR
Trincado (2018)	Depressive symptoms	Individuals with MDD	4 (RCT)	NR	591 (NA)	Placebo or antidepressant alone	SMD= -0.26 (-0.6, 0.08)	NR	NR
Jin (2022)	Perinatal depression	Pregnant women	4 (PC), 3 (CS)	NR	12,881 (2,378)	No supplement	**OR=0.71 (0.59, 0.83)**	74.4% (p=0.001)	NR
**Depression – Serum/RBC folate**									
Jin (2022)	Perinatal depression	Pregnant women	5 (PC)	NA	6,442 (1,742)	Healthy control	**SMD= -0.13 (-0.18, -0.07)**	60.9% (p=0.04)	NR
**Depression – Dietary/ Serum/ RBC folate**									
Bender (2017)	Depression	General population	43 (CC)	NA	35,801 (8,519)	Healthy control	**g= -0.24 (-0.31, -0.16)**	84.9%	p=0.03
**Bipolar disorder – Serum folate**									
Hsieh (2019)	Bipolar disorder	General population	6 (CC)	NA	1,241 (481)	Healthy control	**g= -0.21 (-0.39, -0.03)**	42.2% (p=0.12)	p=0.23
**Autism Spectrum Disorder – Plasma/serum**									
Frustaci (2012)	ASD	Children aged 2-16 years (US, Romania)	5 (CS)	NA	314 (167)	Healthy control	Mean ratio=1.05 (0.97, 1.15)	p=0.3	NR
Gallardo-Carrasco (2021)	ASD	Children aged 2-18 years (Turkey, China)	4 (CC)	NA	299 (148)	Healthy control	SMD= -0.16 (-0.63, 0.32)	74% (p=0.009)	NR
Prades (2022)	ASD	Children aged 4.8 (0.3)–17 (11) years (Norway, US, Romania, Oman, Turkey, China)	12 (CS)	NA	1,665 (710)	Healthy control	MD= 0.05 (-1.28, 1.38)	98.3% (p<.0001)	NR
**Autism Spectrum Disorder – Maternal FA supplement**									

Wang M (2017)	ASD	Overall	5 (PC), 11 (CC)	NR	631,905 (4,514)	No supplement	**RR=0.77 (0.64, 0.93)**	59.7% (p=0.001)	p=0.11
		- Asia	8 (PC, CC)	NR	19,027 (1,581)	No supplement	**RR=0.67 (0.46, 0.97)**	64.9% (p=0.006)	NR
		- Europe	3 (PC, CC)	NR	596,925 (2,258)	No supplement	**RR=0.84 (0.68, 0.99)**	53.5% (p=0.116)	NR
		- US	5 (PC, CC)	NR	15,953 (675)	No supplement	**RR=0.41 (0.17, 0.99)**	68% (p=0.014)	NR
		- Prospective cohort only	5 (PC)	NR	616,911 (2,794)	No supplement	RR=0.90 (0.79, 0.998)	42.6% (p=0.138)	NR
		- Case-control studies only	11 (CC)	NR	14,994 (1,720)	Healthy control	**RR=0.44 (0.26, 0.72)**	66% (p=0.001)	NR
Iglesias Vazquez (2019)	ASD	Children aged 1.5-15 years	6 (PC), 3 (CC)	NR	739,226 (6,396)	No supplement	**OR=0.58 (0.46, 0.75)**	87% (p<.00001)	p<.01
	Motor development	Children aged 11 months-6.5 years	4 (PC)	≥ 400 µg/d	8,805 (NA)	< 400 µg/d	SMD= -0.02 (-0.08, 0.04)	20% (p=0.19)	p=0.19
	Mental development		2 (RCT), 4 (PC)	≥ 400 µg/d	11,302 (NA)	< 400 µg/d	SMD= -0.06 (-0.11, 0.00)	35% (p=0.04)	p=0.04
Guo (2019)	ASD	Mother-offspring pairs (Sweden, Norway) Children aged 3-17 years	2 (PC)	Early pregnancy (median 10.7 weeks or the first 4-8 weeks)	144,865 (2,198)	No supplement	OR=0.92 (0.46, 1.85)	85.5% (p=0.009)	NR
Liu (2022)	ASD	Mother-offspring pairs (Sweden, Norway, Israel) Children aged 4-15 years	3 (PC), 1 (CC)	Before/during pregnancy	693,757 (5,755)	No supplement	**OR=-0.59 (0.39, 0.91)**	p=0.948	NR
**Attention Deficit and Hyperactivity Disorder – Plasma/serum folate**									
Prades (2022)	ADHD	Children aged 7.7 (3.2)-9.2 (1.7) years	5 (CS)	NA	1,121 (537)	Healthy controls	MD= 0.92 (0.01, 1.76)	98.85% (p<.0001)	NR
**Schizophrenia – Serum folate**									
Cao (2016)	Schizophrenia	General population	20 (CC)	NA	2,739 (1,463)	Healthy controls	**SMD= -0.57 (-0.76, -0.37)**	82.8% (p<0.001)	p=0.44
**Schizophrenia – FA supplement**									
Sakuma (2018)	Improvement in total symptoms score	General population	4 (RCT)	FA 2-500 mg/d for 12-16 weeks	121 (NA)	Antipsychotics alone	SMD= -0.25 (-0.62, 0.11)	0%	NR
	Improvement in negative symptoms score	General population	3 (RCT)	FA 2-500 mg/d for 12-16 weeks	104 (NA)	Antipsychotics alone	SMD= -0.20 (-0.59, 0.18)	0%	NR
**Psychosis – Serum folate**									
Firth (2018)	First-episode psychosis	General population	6 (CS)	NA	827 (421)	Healthy control	**g= -0.624 (-1.18, -0.07)**	92.4% (p<.01)	p=0.16
**Obsessive compulsive disorder - Plasma/serum folate**									
Balandeh (2021)	OCD	General population Turkey, Israel	4 (CC)	NA	245 (140)	Healthy control	SMD= -0.23 (-1.01, 0.55)	88% (p<.0001)	p=0.88
Yan (2022)	OCD	General population All Caucasian (Turkey, Iran, Israel)	5 (CC)	NA	309 (172)	Healthy control	SMD= -0.09 (-0.75, 0.58)	87.6% (p=0.000)	NR

* as reported by authors based on visual inspection of funnel plots; **bolded**: statistically significant

AD: Alzheimer's disease; ADHD: attention deficit and hyperactivity disorder; ALS: amyotrophic lateral sclerosis; ASD: autism spectrum disorder; CC: case-control study; CS: cross-sectional study; FA: folic acid; MA: meta-analysis; MD: mean difference; MDD: major depressive disorder; OCD: obsessive compulsive disorder; OR: odds ratio; PC: prospective cohort; PD: Parkinson's disease; RCT: randomized controlled trial; RR: relative risk; SMD: standardized mean difference; SR: systematic review; VaD: vascular dementia; WMD: weighted mean difference

Table S3. Risk of bias assessment of the syntheses examining the association between folate intake/ status and neuropsychiatric disorders

	Domain 1: Study eligibility criteria	Domain 2: Identification and selection of studies	Domain 3: Data collection and study appraisal	Domain 4: Synthesis and findings	Risk of Bias in the Review
Asadi-Pooya A (2021)	⊕	⊕	⊗	⊕	⊗
Balandeh E (2021)	⊕	⊕	⊕	⊕	⊕
Balk E (2007)	⊕	⊗	⊕	⊗	⊗
Barnes J (2014)	⊗	⊗	⊗	⊗	⊗
Bender A (2017)	⊕	⊗	⊗	⊕	⊗
Borges-Vieira JG (2023)	⊕	⊗	⊗	⊗	⊗
Cao B (2016)	⊕	⊗	⊕	⊕	⊗
Castro K (2016)	⊗	⊗	⊕	⊗	⊗
Chia SC (2015)	⊕	⊗	⊗	⊗	⊗
Colapinto CK (2016)	⊕	⊕	⊕	⊕	⊕
Cooper C (2015)	⊕	⊕	⊕	⊗	⊗
de Wilde MC (2017)	⊕	⊗	⊗	⊗	⊗
Dhingra S (2012)	⊕	⊗	⊗	⊗	⊖
Dong B (2020)	⊕	⊗	⊕	⊕	⊗
Ellinson M (2004)	⊕	⊗	⊗	⊕	⊗
Firth J (2017)	⊕	⊕	⊕	⊕	⊕
Firth J (2018)	⊕	⊕	⊗	⊕	⊗
Frazer CJ (2005)	⊕	⊕	⊗	⊗	⊗
Frustaci A (2012)	⊕	⊕	⊕	⊗	⊗
Gabriel F (2023)	⊕	⊗	⊕	⊕	⊗
Gallardo-Carrasco MC (2021)	⊕	⊕	⊕	⊕	⊕
Gil Martinez V (2022)	⊕	⊗	⊗	⊕	⊗
Guo BQ (2019)	⊕	⊕	⊕	⊕	⊕
Harbottle L (2008)	⊕	⊕	⊕	⊕	⊕
Hoxha B (2021)	⊕	⊕	⊗	⊕	⊗
Hsieh YC (2019)	⊕	⊕	⊕	⊕	⊕
Hu N (2023)	⊕	⊕	⊕	⊕	⊕
Huang L (2022)	⊕	⊕	⊕	⊕	⊕
Iglesias Vazquez L (2019)	⊕	⊕	⊕	⊕	⊕
Jia X (2008)	⊕	⊕	⊕	⊕	⊕
Jin X (2022)	⊕	⊕	⊕	⊕	⊕
Liampas IN (2020)	⊕	⊕	⊕	⊕	⊕
Liampas IN (2020)	⊕	⊕	⊕	⊕	⊕
Liu X (2022)	⊕	⊕	⊕	⊕	⊕
Lohner S (2012)	⊕	⊕	⊗	⊕	⊗
Lopes Da Silva S (2014)	⊕	⊗	⊗	⊕	⊗
Main PA (2010)	⊕	⊕	⊗	⊕	⊗
Michelakos T (2013)	⊕	⊕	⊕	⊗	⊗
Morris DW (2008)	⊗	⊕	⊗	⊗	⊗
Murakami K (2010)	⊕	⊗	⊗	⊗	⊗
Murray LK (2019)	⊕	⊗	⊗	⊗	⊗
Nahas R (2011)	⊕	⊗	⊗	⊗	⊗
Perez L (2012)	⊕	⊗	⊕	⊗	⊗
Prades N (2022)	⊕	⊕	⊕	⊕	⊕
Purnell C (2009)	⊕	⊗	⊗	⊗	⊗
Raman G (2007)	⊕	⊗	⊕	⊕	⊗

Ranganathan LN (2009)	⊕	⊕	⊕	⊗	⊗
Sakuma K (2018)	⊕	⊕	⊕	⊕	⊕
Sampaio AC (2021)	⊕	⊗	⊗	⊗	⊗
Sanhueza C (2013)	⊕	⊕	⊕	⊕	⊕
Sarris J (2011)	⊕	⊗	⊗	⊕	⊗
Sarris J (2016)	⊕	⊗	⊗	⊕	⊗
Schefft C (2017)	⊕	⊕	⊕	⊕	⊕
Schneider JA (2006)	⊕	⊖	⊗	⊗	⊗
Shah R (2013)	⊗	⊗	⊗	⊗	⊖
Shen L (2015)	⊕	⊗	⊗	⊕	⊗
Shen L (2015)	⊕	⊕	⊗	⊕	⊗
Sparling TM (2017)	⊕	⊕	⊕	⊕	⊕
Taylor MJ (2010)	⊕	⊕	⊕	⊕	⊕
Trincado J (2018)	⊖	⊖	⊗	⊗	⊗
Trujillo J (2018)	⊕	⊕	⊕	⊕	⊕
Van Dam F (2009)	⊕	⊕	⊕	⊗	⊗
Van der Burg (2021)	⊕	⊕	⊕	⊕	⊕
Vlachos GS (2019)	⊕	⊗	⊗	⊗	⊗
Vogel T (2009)	⊕	⊗	⊗	⊗	⊗
Wald DS (2010)	⊕	⊕	⊕	⊗	⊗
Wang M (2017)	⊕	⊕	⊕	⊕	⊕
Wang Q (2021)	⊕	⊕	⊕	⊕	⊕
Wang Z (2022)	⊕	⊕	⊕	⊕	⊕
Widyadharma IPE (2020)	⊕	⊗	⊕	⊗	⊗
Xie Y (2017)	⊕	⊕	⊕	⊕	⊕
Yan S (2022)	⊕	⊕	⊕	⊕	⊕
Zhang X (2021)	⊕	⊕	⊕	⊕	⊕
Zhong C (2020)	⊕	⊗	⊗	⊕	⊗
High	4	29	30	27	42
Low	68	42	43	46	29
Unclear	1	2	0	0	2
